# Matrix-Product State Skeletons in Onsager-Integrable Quantum Chains

**DOI:** 10.1007/s10955-026-03637-8

**Published:** 2026-06-15

**Authors:** Imogen Camp, Nick G. Jones

**Affiliations:** 1 Rudolf Peierls Centre for Theoretical Physics, University of Oxford, Oxford, UK; 2 St John’s College and Mathematical Institute, University of Oxford, Oxford, UK

## Abstract

Matrix-product state (MPS) skeletons are connected networks of Hamiltonians with exact MPS ground states that underlie a phase diagram. Such skeletons have previously been found in classes of free-fermion models. For the translation-invariant BDI and AIII free-fermion classes, it has been shown that the underlying skeleton is dense, giving an analytic approach to MPS approximation of ground states anywhere in the class. In this paper, we partially expose the skeleton in certain interacting spin chains: the *N*-state Onsager-integrable chiral clock families. We construct MPS that form a dense MPS skeleton in the gapped regions surrounding a sequence of fixed-point Hamiltonians (the generators of the Onsager algebra). Outside these gapped regions, these MPS remain eigenstates, but no longer give the many-body ground state. Rather, they are ground states in particular sectors of the spectrum. Our methods also allow us to find further MPS eigenstates; these correspond to low-lying excited states within the aforementioned gapped regions. This set of MPS excited states goes beyond the previous analysis of ground states on the $N=2$ free-fermion MPS skeleton. As an application of our results, we find a closed form for the disorder parameter in a family of interacting models. Finally, we remark that many of our results use only the Onsager algebra and are not specific to the chiral clock model representation.

## Introduction

In recent decades, significant advances have been made in understanding the ground state physics of one-dimensional quantum many-body systems. While integrability provides an analytical probe of phenomena in special families of Hamiltonians [1, 2], numerical methods related to matrix-product state (MPS) approximations allow us to understand features of the general interacting case [3, 4]. When the many-body Hamiltonian has a spectral gap, the entanglement area law guarantees an MPS approximation to the ground state [5, 6]. Beyond approximations, MPS are also a valuable analytical tool. MPS-solvable Hamiltonians have exact MPS ground states, in the sense that the ground state can be represented with a finite bond dimension in the thermodynamic limit. Such models, including the AKLT chain [7], have led to many insights [6]. For example, MPS approaches have led to a deeper understanding of symmetry fractionalisation, order parameters, and the classification of symmetry-protected topological (SPT) phases in one dimension [8–11].

It is interesting to consider the connection between integrable models and Hamiltonians that admit exact MPS ground states: neither property implies the other, yet both facilitate analytic insights into quantum many-body systems. Simple examples of MPS-solvable Hamiltonian families are the one-parameter “disorder line” of product states in the XY model [12–16], and its MPS-solvable Kramers-Wannier dual [17, 18]. Refs. [19, 20] investigated this question more broadly in families of free-fermion chains (the translation-invariant BDI and AIII classes of the tenfold way [21]). Despite the simple exact-solvability of free fermion models [22], their ground states are generically *not* exact matrix-product states, as their entanglement spectra have infinite rank [19, 23]. However, taking the BDI class for definiteness, we can precisely characterise the subset of this family with exact MPS ground states. Each model in the BDI class corresponds to a Laurent polynomial *f*(*z*), and the model has an exact MPS ground state if and only if $f(z)= z^p g(z)^2 h(z)$ for some integer *p*, polynomial *g*(*z*), and Laurent polynomial *h*(*z*), where $h(z)=h(1/z)$. The sub-family satisfying this condition constitutes the MPS skeleton: a structure underlying the entire phase diagram. In contrast to finding an MPS approximation to the ground state of a general gapped Hamiltonian, the ground states along this skeleton can be represented exactly by an MPS with finite bond dimension in the thermodynamic limit. While lying exactly on the skeleton is fine-tuned, it is dense in the following sense: we can find a sequence of models lying on the skeleton with ground states that give an arbitrarily good approximation to the energy density of a generic ground state in the class. Hence, we have an analytic approach for approximating those ground states in the phase diagram that have an infinite-rank entanglement spectrum [19, 20].

It is natural to ask if there are other families of models where the underlying MPS skeleton can be identified. It is of particular interest to go beyond free-fermionic cases. In this work, we construct MPS skeletons underlying interacting families of Onsager-integrable chiral clock models. These are families of integrable models on chains of *N*-state sites, where the Onsager algebra yields local conserved charges [24–26]. Taking $N=2$, we recover the BDI class (in its spin chain form). Onsager’s 1944 computation of the free energy in the 2D classical Ising model utilised this algebra, and it is thus naturally connected to the 1D quantum transverse-field Ising model and its free-fermion solvability. Nevertheless, other representations of this algebra appear as *N*-state generalisations of the quantum Ising model. For $N>2$, these models are interacting chiral clock models [27–30]. Commutators of the Onsager paramagnet, denoted $A_0$, and the Onsager ferromagnet, denoted $A_1$, generate this algebra. Beyond the superintegrable chiral Potts chain $A_0+\lambda A_1$, the analogue of the transverse-field Ising model, such models remain relatively unexplored. Ref. [26] examines the Hamiltonian family constructed as a linear combination of the generators, denoted $A_k$, while Refs. [31, 32] analyse their SPT physics. Note that in some cases the Onsager generators are considered as a symmetry algebra of some other Hamiltonian, rather than as Hamiltonians themselves [33–36].

**Fig. 1:**
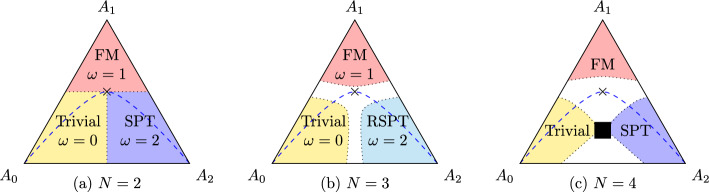
Schematic phase diagrams for the Hamiltonian $H = \alpha A_0+\beta A_1 + \gamma A_2$ with normalisation $\alpha +\beta +\gamma =1$. The winding number of *f*(*z*) is denoted by $\omega$. The trivial phase has $\omega =0$, while $\omega =1$ corresponds to the ferromagnetic (FM) phase. For even *N*, $\omega =2$ gives an SPT; for odd *N*, we instead have a representation-SPT (RSPT). The coloured regions are gapped and connected to fixed-point $A_k$, therefore corresponding to the intersection of the set $\mathcal {S}$ with the phase diagram. The white regions are gapless and outside $\mathcal {S}$. For $N=2$, the phase diagram is well-understood [17, 18]. Diagrams for $N=3,4$ were found numerically in Ref. [31]. For $N=4$, it is unclear whether the trivial and SPT phases have a direct transition at the black square. The dashed line represents a leg of the skeleton parameterised by $H = (1-\lambda )^2A_0 + 2\lambda (1-\lambda )A_1 + \lambda ^2A_2$. For $N=2$, the ground state is an MPS along the entire path. We show that the analogous state is an eigenstate for general *N*, and is the ground state in the gapped regions. The crosses represent points where the MPS construction given in this paper has a singularity; this coincides with a gap closing in the sector $\mathcal {S}.$

As demonstrated in [30], we can express generators of (general) representations of the Onsager algebra as a direct sum of representations of *sl*(2). In principle, we can use this to diagonalise these models, establishing the *form* of the spectrum [26, 30, 37]. In the chiral clock models, these representations are all two-dimensional [38, 39]. Further analysis is required to determine the spectrum from functional equations in the chiral clock case [29, 38–42]—see also Refs. [37, 43, 44] for more recent work on eigenvectors. These eigenvectors are, roughly speaking, found by filling an interacting Fermi sea [38, 40, 45]. While the ground-state phase diagram for the $N=2$ case is well understood [46, 47], we have a far more limited understanding of the phase boundaries for $N>2$. However, we expect gapless regions comprising a sequence of first-order transitions between different Onsager sectors, as occurs when tuning $A_0 + \lambda A_1$ [45, 48, 49]. Numerical results from [31] demonstrate this extended gapless region in a two-parameter phase diagram of $A_0+\lambda A_1 + \mu A_2$ (see also Fig. 1).

For each *N*, we consider the family of Hamiltonians

1 $$\begin{aligned} H_A[\{t_m\},N]= \sum _m t_m A_m \qquad t_m\in \mathbb {R}\ .\end{aligned}$$

For $N=2$, these spin chains are generalised cluster models [46, 50–55]

2 $$\begin{aligned} H_A[\{t_m\},2] {=} {-} \frac{t_0}{4} \sum _j \sigma ^x_j {-} \frac{t_1}{4}\sum _j \sigma ^z_j\sigma ^z_{j+1}- \frac{t_{-1}}{4}\sum _j \sigma ^y_j\sigma ^y_{j+1} {+}\frac{t_2}{4} \sum _j \sigma ^z_{j-1}\sigma ^x_j\sigma ^z_{j+1} {+}\dots \ ,\end{aligned}$$

where $\sigma _j$ are the usual Pauli operators on site *j*. For the chiral clock models, we show below that each $A_m$ is a local Hamiltonian with a range of |*m*| sites (i.e. is supported on $|m|+1$ sites), and we impose that the overall model is finite-range. This means we allow only finitely many non-zero $t_m$.

Within the family (1), we let

3 $$\begin{aligned} \mathcal {S} {=} \{ H_A[\{t_m\},N] \text {~such~that~} H_A \text {~connects~to~some~} A_k \text {~along~a~gapped~path~within~the~family} \}; \end{aligned}$$

i.e., $\mathcal {S}$ is the set of Hamiltonians that are in the gapped regions surrounding each of the Onsager generators $A_k$. The $A_k$ are fixed-point Hamiltonians, with zero-correlation-length ground states [31]. Then, writing $f(z) = \sum _m t_m z^m$, we show that if $f(z) = \pm z^p g(z)^2$, and we are in $\mathcal {S}$, then we can construct an exact MPS ground state except at a measure zero set of points. Thus, we have *an* MPS skeleton for $N>2$, giving a significant generalisation of the $N=2$ results to the interacting case. However, importantly, we note the implication is in one direction, and there may be other Hamiltonians in $\mathcal {S}$ with exact MPS ground states (this is certainly the case for $N=2$, as we have excluded cases with $h(z)\ne \text {const}.$). We also show that this skeleton is dense within $\mathcal {S}$, giving us an analytic approach to approximating the ground state by MPS in this region. A further result of our analysis is the first excited state at zero momentum for models on the skeleton, along with the lowest-energy eigenstate in each non-zero momentum sector, up to the points where these states become degenerate with others in the same sector. This is a direct analogue to the appearance of an exact MPS ground state in the zero momentum sector: usually, we can approximate gapped ground states in finite-momentum sectors to be of this form up to small corrections [56, 57].

As with the spectrum, computing correlations in the ground state of $H_A$ is not straightforward [58]. Remarkable closed formulae exist for the long-distance behaviour of the order and disorder parameters along the line $A_0+\lambda A_1$ [42, 48, 59]. While the class $H_A$ can be analysed using the Bethe ansatz, MPS-solvability gives an alternative approach to computing order parameters in the more general family $H_A$, and we demonstrate this for $H_A = A_0+2 a A_1 + a^2 A_2$.

Many of our results rely only on the algebraic relations, and hence apply in principle to other Onsager-integrable Hamiltonians (subject to some additional assumptions given in Section 2.4). Of course, any unitary transformation applied to the generators $A_k$ will preserve the algebra and the interpretation of the $A_k$ as Hamiltonians. Our results can be simply applied to such cases. For example, in Ref. [26], the authors generate other families of Hamiltonians by combining unitary pivots [31, 60] with Kramers-Wannier duality [61, 62]. It would be interesting to find a model where the *sl*(2) representations have dimension greater than two [38], and to connect to the constructions in Refs. [63–65]. For the remainder of the paper we focus our attention on the chiral clock representations.

We structure the paper as follows. In Section 2, we summarise key properties of our model and state our main results. Section 3 illustrates these results with some explicit examples. Section 4 gives more details of the diagonalisation of Onsager-integrable Hamiltonians. In Section 5, we prove the results of Section 2. We conclude in Section 6 with a discussion of possible directions for future work.

## Key Definitions and Statement of Results

### Onsager-Integrable Hamiltonians

In Ref. [24], Onsager solved the two-dimensional classical Ising model by computing the spectrum of the transfer matrix, giving the exact partition function. Of interest to us are the corresponding quantum Hamiltonians on a chain, which can be determined by taking a strongly anisotropic limit of the two-dimensional classical model. His solution utilised what is now called the Onsager algebra: the infinite-dimensional Lie algebra obeyed by the generators $\{A_l, G_m| l,m \in \mathbb {Z}\}$ given by

4 $$\begin{aligned} [A_l, A_m] = G_{l-m} \quad \quad \quad [G_l, A_m] = \frac{A_{m+l}-A_{m-l}}{2}\quad \quad \quad [G_l, G_m] = 0\ . \end{aligned}$$

We observe that the mappings $A_m \mapsto A_{m+1}, G_l\mapsto G_{l}$ and $A_m \mapsto -A_{m}, G_l\mapsto G_{l}$ both leave Eq. (4) invariant.

From this structure, it follows that $A_0$ and $A_1$ obey the Dolan-Grady conditions [25, 66–68]

5 $$\begin{aligned} \Big [\big [[A_1,A_0],A_0\big ],A_0\Big ]=[A_1,A_0] \qquad \qquad \Big [\big [[A_0,A_1],A_1\big ],A_1\Big ]=[A_0,A_1] \ . \end{aligned}$$

In fact, Eqs. (4) and (5) are equivalent [66, 67], and Eq. (5) remains true replacing $A_0\mapsto A_l$, $A_1\mapsto A_m$ [31]. Ref. [69] demonstrates that these conditions give rise to operators that we rewrite as

6 $$\begin{aligned} R_{m,l} = \frac{1}{4}A_m + \frac{1}{2}G_{m-l}-\frac{1}{4}A_{2l-m}\ , \end{aligned}$$

obeying the commutation relations

7 $$\begin{aligned} {[}R_{m,l}, A_l] = R_{m,l} \quad \quad \quad [R_{m,l}^\dagger , A_l] = -R_{m,l}^\dagger \ . \end{aligned}$$

We can therefore interpret $R_{m,l}$ as ladder operators for $A_l$; this structure ensures that the spectrum of $A_l$ consists of sectors with unit level spacing.

We can construct $A_0$ and $A_1$ that are periodic, *L*-site, $\mathbb {Z}_N$-symmetric chiral clock chains with local Hilbert space dimension *N*, and that obey the Onsager algebra. We will take the chain length *L* to be finite. Moreover, for convenience, we will choose $L=0 \mod N$, which we discuss further in Appendix D. Let us define the single-site “shift” and “clock” operators $X_j$ and $Z_j$ obeying $(X_j)^N = (Z_j)^N = 1$ and $X_jZ_k = \omega ^{\delta _{jk}} Z_kX_j$, where $\omega = e^{2\pi i/N}$. In the *Z*-diagonal basis, with states $|a\rangle$ defined modulo *N*, we have

8 $$\begin{aligned} X_j = \sum _{a_j=0}^{N-1}|a_j-1\rangle \langle a_j| \quad \quad \quad Z_j=\sum _{a_j=0}^{N-1}\omega ^{a_j}|a_j\rangle \langle a_j|. \end{aligned}$$

The first two generators of the Onsager algebra, related by Kramers-Wannier duality, are given by

9 $$\begin{aligned} \begin{aligned} A_0 = -\frac{1}{N} \sum _{j=1}^L \sum _{m=1}^{N-1} \alpha _m X_j^m \quad \quad \quad A_1 = -\frac{1}{N} \sum _{j=1}^L \sum _{m=1}^{N-1} \alpha _m (Z_{j-1}^\dagger Z_j)^m \end{aligned} \end{aligned}$$

for complex couplings $\alpha _m = (1-\omega ^m)^{-1}$. In the *Z*-diagonal basis, the eigenstates of $A_1$ are $\{|a_1a_2\dots a_L\rangle \}$ and the eigenstates of $A_0$ are $\{|v_1^{(n_1)}v_2^{(n_2)}\dots v_L^{(n_L)}\rangle \}$ for

10 $$\begin{aligned} |v_j^{(n)}\rangle = \frac{1}{\sqrt{N}}\sum _{a_j=0}^{N-1}\omega ^{-na_j}|a_j\rangle \ . \end{aligned}$$

The ground state of $A_0$ is the unique state $|v_1^{(0)}v_2^{(0)}\dots v_L^{(0)}\rangle$, while $A_1$ has the *N* ferromagnetic ground states $\{|\tau _a\rangle = |aa\dots a\rangle \}$ for $a \in \{0,1,\dots ,N-1\}$. These states all have the same energy $E_0 = -L(N-1)/2N$.

The remaining generators can be found using either the commutation relations Eq. (4) or the pivot procedure described in Ref. [31], utilising the relation

11 $$\begin{aligned} e^{\beta A_m} A_l e^{-\beta A_m} = \cosh ^2\left( \frac{\beta }{2}\right) A_l + \sinh (\beta ) G_{m-l}-\sinh ^2\left( \frac{\beta }{2}\right) A_{2m-l} \qquad \beta \in \mathbb {C}\ . \end{aligned}$$

Consequently, all $A_l$ for even (odd) *l* are related by a unitary transformation to $A_0$ ($A_1$), since12for $U_m = e^{-i\pi A_m}$. Hence, all of these Hamiltonians have the same ground state energy $E_0$, and the ground state $|\psi _l\rangle$ of $A_l$ is unique (*N*-fold degenerate) for even (odd) *l*. Additionally, by considering the light cone of $(U_1 U_0)^k$, it follows that each $A_m$ has interaction range at most |*m*|; our generators are therefore local operators in this representation. We emphasise that while any $A_l$ or $G_m$ could be calculated using this procedure, for $N>2$, a general closed-form expression is not known; however, for $A_{-1}$ and $A_2$, closed formulae have been determined [26, 31, 33].

Recall that we work with the family of Hamiltonians of the form

13 $$\begin{aligned} H_A[\{t_m\}, N] = \sum _m t_m A_m \qquad t_m \in \mathbb {R} \ . \end{aligned}$$

It is convenient to define the corresponding Laurent polynomial

14 $$\begin{aligned} f(z) = \sum _m t_m z^m\ . \end{aligned}$$

As shown in Results 1 and 2 of Ref. [19], for $N=2$, if we can write

15 $$\begin{aligned} f(z)=\pm z^p g(z)^2 \quad \quad \quad \text {with}\quad \quad \quad g(z) = \sum _{k=0}^d s_k z^k \end{aligned}$$

and some integer *p*, then we can write the ground state as an exact MPS.^1^ Hamiltonians parameterised by such coefficients $\vec {s} = (s_0,s_1, s_2, \dots , s_d)$ are said to “lie on the skeleton”. In fact, for $N=2$, the ground state can be expressed as an MPS if and only if $f(z)= z^p g(z)^2 h(z)$, where *h*(*z*) is any Laurent polynomial obeying $h(z) = h(1/z)$. This is loosely motivated by noting that, as discussed in Section 4, the spectrum of $H_A[\{t_m\}, N]$ involves terms with coefficients $\sim \sqrt{f(z)f(1/z)}$. For Hamiltonians corresponding to Eq. (15), these terms simplify to $\sim g(z)g(1/z)$; the lack of a square-root is suggestive of simplifications in the analysis. We aim to generalise the results of Ref. [19] by demonstrating that, for $N>2$, Hamiltonians satisfying Eq. (15) will have exact MPS ground states in certain gapped regions. However, we will *not* claim that such a condition is *necessary* for the ground state to be an exact MPS.

As illustrated in Fig. 1, the $N>2$ phase diagrams have extended gapless regions; this contrasts with the $N=2$ case. We will find that, on the $N>2$ skeletons, we can construct MPS eigenstates analogous to the $N=2$ ground state except at a measure-zero set of points. However, we will find that these eigenstates are only ground states in gapped regions containing fixed-point Hamiltonians $A_l$. We represent these by the coloured regions in Fig. 1. It is therefore convenient to define the set $\mathcal {S}$ as the set of Hamiltonians in the class $H_A$ that lie within such gapped regions, as in Eq. (3).

Finally, we note that the structure of the Onsager algebra imposes that

16 $$\begin{aligned} (A_n+G_n)|\psi _0\rangle \propto |\psi _0\rangle \quad \quad \quad (A_n+G_n)|\phi ^{(P)}_{E_0+1/N, \, p=0}\rangle \propto |\phi ^{(P)}_{E_0+1/N, \, p=0}\rangle \ , \end{aligned}$$

where $|\psi _0\rangle$ is the ground state of $A_0$ and

17 $$\begin{aligned} |\phi ^{(P)}_{E_0+1/N, \, p=0}\rangle = \frac{1}{\sqrt{L}}\sum _{j=1}^L e^{-i P j} Z_j^\dagger |\psi _0\rangle \quad \quad \quad P \in \bigg \{0, \frac{2\pi }{L}, \frac{4\pi }{L} , \dots , \frac{2(L-1)\pi }{L}\bigg \}\ . \end{aligned}$$

We will use these results in Section 5.1, and they are proved in Appendix A using the ladder operators Eq. (7).

### Remarks on the Phase Diagram

The family of models, $H_A$, has a $D_{2N}=\mathbb {Z}_N\rtimes \mathbb {Z}_2^{\textrm{CPT}}$ symmetry. The $\mathbb {Z}_N$ generator is $\prod _j X_j$, while the $\textrm{CPT}$ symmetry combines charge conjugation, lattice inversion, and complex conjugation [31]. For all *N*, the $A_{2k+1}$ are symmetry-breaking phases, with *N*-fold ground-state degeneracy. In the symmetric phases we have a unique ground state, and it is of interest to consider its SPT class [9]. For odd *N*, $D_{2N}$ has no SPT phases, and each $A_{2k}$ is in the trivial symmetric phase. For even *N*, $A_{4k}$ is trivial while $A_{4k+2}$ is a non-trivial $D_{2N}$ SPT; this was shown using charge pumps in Ref. [32]. For odd *N*, $A_2$ has some SPT-like features, and, while trivial, the nearby region is labelled a representation-SPT (RSPT) [31, 70, 71], due to a non-trivial linear symmetry-fractionalisation in the leading entanglement levels.

The above analysis relates to the SPT class when allowing arbitrary symmetric perturbations to our Hamiltonian. In the $N=2$ case, the BDI class has an integer topological invariant, coinciding with the winding number of *f*(*z*). This cannot change without a phase transition, provided we stay within the BDI class of free-fermion models [46]. In fact, the set $\mathcal {S}$ inside the family $H_A$ also has the same integer invariant. As we discuss in Section 4, zeros of *f*(*z*) on the unit circle correspond to a bulk gap closing within $\mathcal {S}$. Unlike the $N=2$ case, we can leave $\mathcal {S}$ without a zero crossing the unit circle due to a first order transition to a different sector. However, if we remain in $\mathcal {S}$, we cannot have a zero of *f*(*z*) cross the unit circle, and so the winding number is invariant. This is one way to understand the distinct symmetric phases appearing for $N=3$ in Fig. 1: they have the same SPT invariant, but different winding numbers. Thus, $\mathcal {S}$ consists of disjoint gapped regions around each of the $A_k$, each dual under Kramers-Wannier and unitary pivots.

### Main Results

Here, we state our main results; these will be proved in Section 5. We characterise exact eigenstates of Hamiltonians on connected networks $f(z) = \pm z^p g(z)^2$ within the family Eq. (13) and show that they are ground states in certain regions of the phase diagram. Moreover, these states can be written in exact MPS form.

To write down the closed form of the wavefunction, we must first compute a collection of coefficients $\{b_k\}$ from the coefficients $\{s_k\}$ of $g(z) = \sum _k s_k z^k$. This can be done algorithmically [19], which will be discussed in Section 5.1. We note that this algorithm is identical to the Schur-Cohn algorithm [72, 73], which is used to determine the distribution of the roots of a polynomial with respect to the unit circle. Furthermore, we require the fixed point ground states $|\psi _p^\pm \rangle$ of $\pm A_p$. For the clock models, these can be written in exact MPS form using the procedure given in Ref. [31], and in the symmetry-breaking case the result holds for any of the *N* symmetric ground states $|\psi _p^{(i)}\rangle$.

#### Result 1

(Exact eigenstate) If we can express the Laurent polynomial in the form $f(z) = \pm z^p g(z)^2$ for $p \in \mathbb {Z}$ and

18 $$\begin{aligned} g(z) = \sum _{k=0}^d s_k z^k\ , \end{aligned}$$

then the corresponding Hamiltonian has the eigenstate

19 $$\begin{aligned} |\varphi \rangle = \textrm{M}^{(d)} \textrm{M}^{(d-1)}\dots \textrm{M}^{(1)} |\psi _p^\pm \rangle \ . \end{aligned}$$

Here, $\textrm{M}^{(k)}=\exp (\mp \beta _k A_{p+k})$ for $\beta _k = 2 arctanh (b_k)$, and $|\psi _p^\pm \rangle$ is the ground state of the Hamiltonian $\pm A_p$. Note this breaks down if any $|b_k|=1$.

The states $|\varphi \rangle$ can be expressed in MPS form,^2^ and these Hamiltonians therefore constitute the MPS skeleton; however, a measure-zero set of points, where any $|b_k|=1$, must be excluded. We prove this result in Section 5.1.

#### Result 2

(Exact ground state) For all vectors $\vec {s} = (s_0, \dots , s_d)$ that do not give any $|b_k|=1$, the eigenstate $|\varphi \rangle$ is the ground state of $H_A(\vec {s})$ when $H_A(\vec {s})\in \mathcal {S}$.

This follows straightforwardly from the perturbative arguments we provide in Section 5.2. Note that $|b_k|=1$ occurs in gapless models (see Appendix E), but can also arise for Hamiltonians in $\mathcal {S}$. In such cases, the method used to construct the MPS breaks down, but we can argue that we have an exact MPS ground state by considering a limit of models, lying in $\mathcal {S}$ and on the skeleton, where we can explicitly construct the ground state; Refs. [19, 20] give related discussions. In the $N=2$ case, the distinct methods of Ref. [20] prove the existence of MPS ground states for $f(z)=\pm z^p g(z)^2$ with no restrictions on the $\{b_k\}$.

Although we cannot generally express $f(z)=\pm z^p g(z)^2$ for a finite polynomial *g*(*z*), we can always define and truncate an appropriate “square-root” series at some finite power *D* of *z* [19, 20]. This gives an approximate Hamiltonian $H_A^{(D)}$ corresponding to the Laurent polynomial $f_D(z) = \pm z^k g_D(z)^2$ (note $k\ne p$ in general). For example, the chiral Potts model $A_0 + \lambda A_1$, has Laurent polynomial $f(z) = 1+\lambda z$. This can be approximated by

20 $$\begin{aligned} f_{3}(z) = g_{3}(z)^2 = 1+\lambda z + \frac{5}{64} \lambda ^4 z^4 - \frac{1}{64}\lambda ^5 z^5+\frac{1}{256}\lambda ^6 z^6 \ ,\end{aligned}$$

for $g_{3}(z) = 1+\lambda z/2 -\lambda ^2z^2/8+\lambda ^3z^3/16$. Note that $H_A^{(3)}$ is then the chiral Potts model, plus a “small” perturbation by longer-range models. The ground state of the Hamiltonian $H_A^{(D)}$ can be used to approximate the ground state of $H_A$. Indeed, the skeleton constitutes a dense subset of our family of Hamiltonians in the following sense.

#### Result 3

(The skeleton is dense) Consider a general chiral clock Hamiltonian $H_A$ of the form Eq. (13), with ground state $|\varphi \rangle$, and let $\mathcal {E}_A$ be the corresponding ground state energy density of . We can define a Hamiltonian $H_A^{(D)}$ on the skeleton with ground state $|\varphi _D\rangle$, such that for any fixed $\varepsilon >0$ and sufficiently large *D* and *L*, we find that the energy density $\mathcal {E}_A^{(D)}$ of $|\varphi _D\rangle$ with respect to  satisfies . Analogous statements hold for the ground state in any fixed sector.

The proof is given in Section 5.3. Heuristically, this tells us that the ground state of any Hamiltonian in $\mathcal {S}$ can be approximated by a sequence of states of the form Eq. (19). Ref. [19] gives a derivation of the scaling dimension of the disorder operator in the Ising conformal field theory using such a path of skeleton states. We expect that this can be made precise in the thermodynamic limit using the results of [74].

#### Result 4

(More exact eigenstates) For even *p*, Hamiltonians with Laurent polynomials $f(z) = z^p g(z)^2$ have the exact eigenstates

21 $$\begin{aligned} |\chi _p^{(P)}\rangle = \textrm{M}^{(d)} \textrm{M}^{(d-1)}\dots \textrm{M}^{(1)} |\phi ^{(P)}_{E_0+1/N,\,p}\rangle \ , \end{aligned}$$

where

22 $$\begin{aligned} |\phi ^{(P)}_{E_0+1/N, \,p}\rangle = \frac{1}{\sqrt{L}}\sum _{j=1}^L e^{-i P j} (U_1 U_0)^{p/2} Z_j^\dagger |\psi _0^+\rangle , \quad \quad P \in \bigg \{0, \frac{2\pi }{L}, \frac{4\pi }{L} , \dots , \frac{2(L-1)\pi }{L}\bigg \}\ . \end{aligned}$$

For $f(z) = -z^p g(z)^2$ and even *p*, an equivalent set of eigenstates exist where we replace $Z_j^\dagger \mapsto Z_j$ and $|\psi _0^+\rangle \mapsto |\psi _0^-\rangle$.

This result is proved in Section 5.4, where we also discuss the necessity of even *p* in $f(z)=\pm z^pg(z)^2$ for this to hold. These states can also be represented in exact MPS form.

### Further Remarks

We note that Result 1 can be proved if our model obeys the Onsager algebra and when $|\psi _p^\pm \rangle$ is unique.^3^ For $|\varphi \rangle$ to have an exact MPS representation, we additionally require that $A_0$ and $A_1$ have exact MPS ground states and are finite-range. Moreover, Result 2 requires that our model obeys a finite-dimensional Onsager algebra and that each $A_p$ is gapped. Result 3 holds generally for any finite-dimensional representation of the Onsager algebra, provided that the energy density remains finite in the thermodynamic limit. Result 4, however, is specific to the clock models. Finally, for brevity, we will henceforth write $|\psi _p^+\rangle$ as $|\psi _p\rangle$.

We remark that, just as the $N=2$ case can be viewed as a family of free-fermion models, for $N>2$, our family $H_A$ has a dual parafermionic form [75, 76]. Many of our results will carry over simply to the parafermion picture, although care must be taken with defining parafermionic MPS [77]. Following our discussion about the winding number in Section 2, it would be interesting to understand if there is a connection between this winding and the number of edge modes [76].

## Examples

In this section, we motivate our results by deriving Result 1, Result 2, and Result 4 explicitly for the simplest non-trivial case. We then demonstrate how the ground state can be expressed as an exact MPS. Finally, we show how this result can be used to evaluate the disorder operator on the skeleton, generalising known results for $N=2$ to any even integer *N*.

### Eigenstates for $d=1$

Here, we illustrate our results in the case of $d=1$ using a variation of the method used in the proof for general *d*. The initial steps of the argument are illustrative of the general method, but we are able to make certain computations explicit in this particular case. We also utilise a known expression for the ground state energy in $\mathcal {S}$ that bypasses the perturbative argument of Result 2.

Let us consider the $d=1$ Hamiltonian

23 $$\begin{aligned} H_A = A_{0} + 2 a A_1 + a^2 A_2\ , \end{aligned}$$

which has $p=0$ and $g(z) = 1+az$. The non-interacting $N=2$ case of $H_A$ was studied in detail in Refs. [17, 18].

Defining $\tilde{H}_A = e^{\beta A_1}H_A e^{-\beta A_1}$ for $\beta = 2\text { arctanh}\left( a\right)$, the pivot relation Eq. (11) gives

24 $$\begin{aligned} \tilde{H}_A = (a^2+1)A_{0} + 2a(A_1+G_1)\ . \end{aligned}$$

Consider the ground state $|\psi _0\rangle$ of $A_0$, which—in the chiral clock representation—can be written as the product state [31]

25 $$\begin{aligned} |\psi _0\rangle = |v_1^{(0)}v_2^{(0)}\dots v_L^{(0)}\rangle \quad \quad \quad \text {where}\quad \quad \quad |v_j^{(0)}\rangle = \frac{1}{\sqrt{N}} \sum _{a_j=0}^{N-1} |a_j\rangle \ , \end{aligned}$$

and the translationally invariant basis of single-particle excitations $\{|\phi _{E_0+1/N,\, p=0}^{(P)}\rangle \}$ labelled by momentum *P*. We have

26 $$\begin{aligned} A_{0} |\psi _{0}\rangle = E_0|\psi _{0}\rangle \quad \quad \quad A_{0} |\phi _{E_0+1/N,\, p=0}^{(P)}\rangle = \left( E_0+\frac{1}{N}\right) |\phi _{E_0+1/N,\, p=0}^{(P)}\rangle \ . \end{aligned}$$

Moreover, from Eq. (16), we have

27 $$\begin{aligned} (A_1+G_1) |\psi _{0}\rangle \propto |\psi _0\rangle \quad \quad \quad (A_1+G_1) |\phi _{E_0+1/N,\, p=0}^{(P)}\rangle \propto |\phi _{E_0+1/N,\, p=0}^{(P)}\rangle \ . \end{aligned}$$

The states $|\psi _{0}\rangle$ and $|\phi _{E_0+1/N,\, p=0}^{(P)}\rangle$ are therefore eigenstates of $\tilde{H}_A$. In fact, we can show $(A_1+G_1) |\psi _{0}\rangle = 0$ by noting that there exists an operator $\mathcal {W}$ satisfying both $\mathcal {W}|\psi _0\rangle =|\psi _0\rangle$ and $\{\mathcal {W}, A_{2k+1}\}=0$ [40].^4^ Thus, reversing the transformation, we have

28 $$\begin{aligned} H_A|\varphi \rangle = (a^2+1) E_0 |\varphi \rangle \quad \quad \quad \text {and} \quad \quad \quad H_A|\chi _{p=0}^{(P)}\rangle \propto |\chi _{p=0}^{(P)}\rangle \end{aligned}$$

for

29 $$\begin{aligned} |\varphi \rangle = e^{-\beta A_1}|\psi _0\rangle , \quad \quad \quad |\chi _{p=0}^{(P)}\rangle = e^{-\beta A_1} |\phi _{E_0+1/N,\, p=0}^{(P)}\rangle \ . \end{aligned}$$

Therefore, for all *a*, $|\varphi \rangle$ and $\{|\chi _{p=0}^{(P)}\rangle |P\in \{0, 2\pi /L,\dots ,2(L-1)\pi /L\}\}$ are eigenstates of $H_A$. The state $|\varphi \rangle$ has energy $\varepsilon _\varphi =(a^2+1) E_0$. From Refs. [26, 30], we know that this is the ground state energy provided $H_A \in \mathcal {S}$. Thus, for $d=1$, Result 1 and Result 2 hold.

### Ground State as an MPS for $d=1$

Let us write

30 $$\begin{aligned} e^{-\beta A_1} = \prod _{j=1}^L U_{j, j+1}(\beta )\ , \end{aligned}$$

**Fig. 2:**
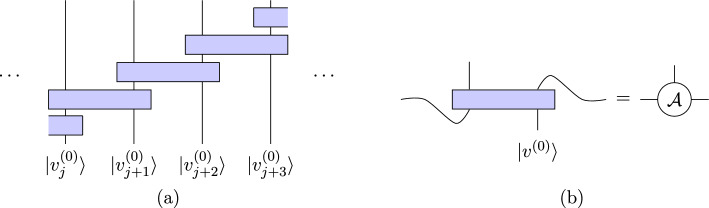
(a) The circuit diagram for the $d=1$ ground state. This can be interpreted as an MPO acting on a product state, giving an MPS. The blue boxes represent each unitary gate $U_{j,j+1}$, and the $|v_j\rangle$ are the single-site states. (b) The MPS tensor from Eq. (33).

where31are commuting operators. The ground state $|\varphi \rangle$ can therefore be represented by the circuit in Fig. 2. This can be interpreted as a matrix-product operator (MPO) acting on a product state, giving an MPS. We obtain the (unnormalised) MPS form

32 $$\begin{aligned} \begin{aligned} |\varphi \rangle&= \sum _{a_1 \dots a_L = 0}^{N-1}\left( \prod _{j=1}^L U_{j-1, j}(\beta ) \right) |a_1 a_2 \dots a_L\rangle \\&= \sum _{a_1 \dots a_L = 0}^{N-1} \text {Tr}\left[ \mathcal {A}^{(a_1)}_1 \mathcal {A}^{(a_2)}_2 \dots \mathcal {A}^{(a_L)}_L\right] |a_1 a_2 \dots a_L\rangle \end{aligned} \end{aligned}$$

with the MPS tensors

33 $$\begin{aligned} \begin{aligned} \left( \mathcal {A}_j^{(a_j)}\right) _{\sigma _{j-1}, \sigma _j}&= \delta _{\sigma _{j-1} a_j} \exp \left( \frac{\beta }{N}\sum _{m=1}^{N-1} \alpha _m \omega ^{-m(a_{j}-\sigma _{j})}\right) \\&=\delta _{\sigma _{j-1} a_j} \exp \left( \frac{\beta (N-1)-2\beta ((a_j-\sigma _j)\mod N)}{2N}\right) \end{aligned} \end{aligned}$$

of bond dimension $\chi = N$.

### Disorder Operator on the $d=1$ Skeleton

For even *N*, we define the unitary disorder operator

34 $$\begin{aligned} \mu _k = \prod _{j=-\infty }^k X_j^{N/2}\ . \end{aligned}$$

For $N=2$, and for *g*(*z*) such that all $|b_k|<1$, the expectation of the disorder operator in the ground state is [19]

35 $$\begin{aligned} \langle \mu _k\rangle _{N=2} = \prod _{l=1}^d (1-b_l^2)^{l/2}\ . \end{aligned}$$

For $g(z)=1+az$, with $|a|<1$, this becomes $\langle \mu _k\rangle _{N=2} = \sqrt{1-a^2}$. Here, we outline how this $d=1$ result generalises to any even *N*. Full details of the calculation are given in Appendix B.

We aim to evaluate^5^

36 $$\begin{aligned} \langle \mu _k\rangle = \frac{\langle \varphi |\mu _k|\varphi \rangle }{\langle \varphi |\varphi \rangle } = \frac{\langle \psi _0|e^{-\beta A_1}\mu _ke^{-\beta A_1}|\psi _0\rangle }{\langle \psi _0|e^{-2\beta A_1}|\psi _0\rangle }\ . \end{aligned}$$

If we define37then we can use the commutation relations $X_j Z_k = \omega ^{\delta _{jk}}Z_k X_j$ and the fact that $\mu _k |\psi _0\rangle = |\psi _0\rangle$ to express the numerator as

38 $$\begin{aligned} \langle \psi _0|e^{-\beta A_1}\mu _ke^{-\beta A_1}|\psi _0\rangle =\langle \psi _0|e^{-2\beta A_1} \prod _{\begin{array}{c} m=1 \\ m \text { odd} \end{array}}^{N-1}U_{k,k+1}^{(m)}(-2\beta ) |\psi _0\rangle \ . \end{aligned}$$

If we write $|\psi _0\rangle$ in terms of the states $|a\rangle$ that are diagonal in the *Z*-basis

39 $$\begin{aligned} |\psi _0\rangle = \frac{1}{\sqrt{N^L}}\sum _{a_1\dots a_L =0}^{N-1} |a_1 \dots a_L\rangle \ , \end{aligned}$$

then $\langle \mu _k\rangle$ can be written as the ratio of two sums over configurations of states in the *Z*-basis. We can evaluate these expressions with a transfer matrix method, writing

40 $$\begin{aligned} \langle \mu _k\rangle = \frac{\text {Tr}(T^{L-1} B)}{\text {Tr}(T^L)} \end{aligned}$$

for two matrices *T* (the transfer matrix) and *B*. We find that these matrices are circulant, and so their spectra can be determined from standard results [78]. In the thermodynamic limit $L\rightarrow \infty$, the largest eigenvalue of *T* dominates, and we obtain

41 $$\begin{aligned} \langle \mu _k\rangle = \sqrt{1-a^2} \end{aligned}$$

for all even *N*, as desired.

## The Spectrum of Onsager-Integrable Chiral Clock Chains

In this section, we summarise how the Onsager algebra can be used to examine the structure of the spectrum. As stated in Result 2, the eigenstate $|\varphi \rangle$ is the ground state of Hamiltonians in the region $\mathcal {S}$, defined by Eq. (3). As this region is determined by the spectral gap of the Hamiltonians, it is important to understand some properties of their spectra. The reader may wish to skip this section on a first read, noting only Eq. (46) which is used in Section 5.5.

Firstly, we note that chiral clock Hamiltonians commute with the translation operator and the $D_{2N}=\mathbb {Z}_N\rtimes \mathbb {Z}_2^{\textrm{CPT}}$ generators described in Section 2. The Hamiltonian can therefore be block-diagonalised into sectors labelled by the quantum numbers of momentum and $\mathbb {Z}_N$-charge, denoted *P* and *Q* respectively. Additionally, as discussed in Refs. [30, 37–39, 79], the Hamiltonians can be further block-diagonalised into Onsager sectors, with multiple Onsager sectors per (*P*, *Q*) sector. We define the “ground-state Onsager sector”, lying within the (0, 0) sector, to be the Onsager sector containing the ground state of $A_0$.

As discussed in Refs. [26, 30], given a finite-dimensional representation of the Onsager algebra, it is possible to write $\sum _{m=-n}^n \alpha _m A_m = 0$ for some integer *n* and coefficients satisfying $\alpha _m = \pm \alpha _{-m}$; determining these coefficients is, however, non-trivial. Nevertheless, one can define $z_{\mp k} = e^{\pm i m \theta _k }$ as the roots of the polynomial $\mathfrak {h}(z) = \sum _{m=-n}^n \alpha _m z^{m+n}$. The generators of any finite-dimensional representation of the Onsager algebra can then be rewritten, up to sector-dependent additive constants, with the invertible transformation^6^

42 $$\begin{aligned} A_m = \frac{1}{2} \sum _{k=1}^n e^{-im\theta _k} E_k^+ + e^{im\theta _k}E_k^-\quad \quad \quad G_m = \frac{1}{2}\sum _{k=1}^n \left( e^{-im\theta _k} - e^{im\theta _k}\right) H_k\ , \end{aligned}$$

where the Onsager algebra imposes that

43 $$\begin{aligned} {[}E_j^+,E_k^-]=\delta _{jk}H_k\quad \quad \quad {[}H_j,E_k^\pm ]=\pm 2\delta _{jk}E_k^\pm \ . \end{aligned}$$

The operators $\{E_j^\pm , H_k\}$ can therefore be interpreted as *sl*(2) generators. The roots of $\mathfrak {h}(z)$ lie on the unit circle to ensure the self-adjointness of our Hamiltonian, and they are also sector-dependent.

In general, determining the *n* values $\{\theta _k\}$ is difficult and must be done by solving functional equations [28–30, 40, 41]. (These are referred to as BAMP polynomials in Ref. [42] and chiral Potts polynomials in Ref. [80].) However, without calculating the values $\theta _k$, we can easily establish the *structure* of the spectra of Onsager-integrable Hamiltonians by writing

44 $$\begin{aligned} H_A = \sum _m t_m A_m = \sum _m \sum _{k=1}^n t_m \bigg [\cos (m\theta _k) J_x^k + \sin (m\theta _k) J_y^k\bigg ]\ , \end{aligned}$$

where we have defined $E^\pm _k = J_x^k \pm i J_y^k$. This can be diagonalised by rotating in the $(J_x,J_y)$ plane to give

45 $$\begin{aligned} \begin{aligned} H_A&= \sum _{k=1}^n \sqrt{\sum _{l,m}t_l t_m \cos \left( (m-l)\theta _k\right) } J^k_{x, \text {new}} \\  &= \sum _{k=1}^n \sqrt{f(e^{i\theta _k})f(e^{-i\theta _k})}J^k_{x, \text {new}}\\&= \sum _{k=1}^n g(e^{i\theta _k}) g(e^{-i\theta _k}) J^k_{x, \text {new}}\ , \end{aligned} \end{aligned}$$

where the final line follows only if we are on the skeleton, where $f(z)=\pm z^p g(z)^2$. Given that we know the eigenvalues of the spin-operators $J^k_{x, \text {new}}$, the form of the spectrum in each sector, up to additive constants, is

46 $$\begin{aligned} \begin{aligned} \varepsilon _A(\{m_k\})&= \sum _{l,m}s_l s_m\sum _{k=1}^n m_k \cos ((l-m)\theta _k) \end{aligned} \ . \end{aligned}$$

Here, each of the $m_k$ can take values from $\{-S, \dots , S-1, S\}$ for the spin-*S* representation of *sl*(2); in the clock models we have $S=1/2$ [30]. Again, we emphasise that this form is up to sector-dependent additive constants.

For chiral-clock Hamiltonians $H_A\in \mathcal {S}$, the ground state lies in the “ground-state Onsager sector”. This follows from perturbative arguments and pivoting and duality relations. In this sector, the $\{\theta _k\}$ values can be calculated [28, 29, 38] and have multiplicity one.^7^ The ground state energy density for chiral clock Hamiltonians $H_A \in \mathcal {S}$ in the thermodynamic limit is then given by [28]

47 $$\begin{aligned} \varepsilon _0 = -\frac{1}{2\pi }\sum _{l,m} s_l s_m \int _0^{\pi (1-1/N)} \cos \Big ((l-m)\theta \Big )\,\textrm{d}\zeta \end{aligned}$$

where

48 $$\begin{aligned} \tan (\theta /2) = \left( \frac{\sin (\zeta )}{\sin (\zeta +\pi /N)}\right) ^{N/2} \end{aligned}$$

and $0\le \theta \le \pi$. Since we have $f(e^{\pm i \theta })$ in Eq. (45), this range of $\theta$ covers the unit circle. Thus Eq. (45) connects gapless modes in this sector to zeros of $f(e^{i \theta })$ on the unit circle. This means that, even when we do not have a first-order transition to a different sector, if we tune a zero to the unit circle, $H_A$ cannot remain in $\mathcal {S}$. This is consistent with previous analysis of the superintegrable chiral Potts line [81].

As mentioned above, some results for the eigenvectors are also known [37, 38, 40, 43, 44]. These are typically given for the model $A_0 + \lambda A_1$, but we expect they could be generalised. However, this would correspond in the $N=2$ case to knowledge of the diagonal free-fermionic modes. For $N=2$, going from the mode representation to the MPS representation is non-trivial [19], and our simple construction of the MPS eigenstates in these interacting models gives a new perspective and a direct approach that is mostly decoupled from the exact solutions above.

## Analysis

In this section, we prove the results given in Section 2. Given that Hamiltonians with $f(z) = \pm z^p g(z)^2$ are all related to the Hamiltonian with $f(z) = \pm g(z)^2$ via some combination of Kramers-Wannier duality and unitary transformations, we first prove our results for $p=0$.

### Proof of Result 1

We begin by noting that the pivot relation Eq. (11), following directly from the Onsager algebra, means that abstractly

49 $$\begin{aligned} \begin{aligned} \tilde{H}_A&= e^{\beta _1 A_1} \dots e^{\beta _d A_d} H_A e^{-\beta _d A_d} \dots e^{-\beta _1 A_1}\\&= \sum _\alpha a_\alpha A_\alpha + g_\alpha G_\alpha \end{aligned} \end{aligned}$$

has coefficients $\{a_\alpha , g_\alpha \}$ independent of our choice of representation. Therefore, if we find the explicit form of $\tilde{H}_A$ in one representation, the form must hold for all representations provided we do not have any representation-specific cancellations of the generators.^8^

Thus, we will study a particular (free-fermion) representation below. Define the Majorana fermions

50 $$\begin{aligned} \gamma _j = \left( \prod _{k=1}^{j-1}X_k\right) Y_j \quad \quad \quad \tilde{\gamma }_j = \left( \prod _{k=1}^{j-1}X_k\right) Z_j\ , \end{aligned}$$

where $X_j, Y_j, Z_j$ reduce to the usual $2\times 2$ Pauli matrices. A representation of the Onsager algebra has generators

51 $$\begin{aligned} A_k = \frac{1}{4}\sum _{n=1}^L i \tilde{\gamma }_n \gamma _{n+k} \quad \quad \quad G_k = \frac{1}{8}\sum _{n=1}^L \tilde{\gamma }_n\tilde{\gamma }_{n+k} -\gamma _{n}\gamma _{n+k}\ . \end{aligned}$$

This is a slightly different $N=2$ representation of the Onsager algebra, related to our definition of the $N=2$ chiral clock model by a unitary transformation (see Section 2.1). Using results from Ref. [19], Hamiltonians in this representation with $f(z) = z^p g(z)^2$ can be expressed as

52 $$\begin{aligned} H_A = \frac{1}{8} \sum _{n=1}^L \left( \Omega _n\Gamma _n - 2|\vec {s}|^2\right) \ , \end{aligned}$$

where

53 $$\begin{aligned} \Gamma _n = \sum _{\alpha =0}^d s_\alpha \left( \gamma _{n+\alpha } - i \tilde{\gamma }_{n-\alpha }\right) \quad \quad \quad \Omega _n = \sum _{\alpha =0}^d s_\alpha \left( \gamma _{n+\alpha } + i \tilde{\gamma }_{n-\alpha }\right) \ . \end{aligned}$$

In contrast with the approach taken in Ref. [19], we treat $\Omega _n$ and $\Gamma _n$ as separate objects rather than writing $\Omega _n = \Gamma _n^\dagger$ and considering only transformations on $\Gamma _n$.

Begin by defining $\textrm{M}^{(k)}$ as in Result 1. We wish to compute

54 $$\begin{aligned} \tilde{H}_A = \textrm{M}^{(1)^{-1}}\dots \textrm{M}^{(d)^{-1}} H_A \textrm{M}^{(d)}\dots \textrm{M}^{(1)}\ , \end{aligned}$$

which can be done by taking

55 $$\begin{aligned} \Gamma _n \mapsto \tilde{\Gamma }_n = \textrm{M}^{(1)^{-1}}\dots \textrm{M}^{(d)^{-1}} \Gamma _n \textrm{M}^{(d)}\dots \textrm{M}^{(1)} \end{aligned}$$

56 $$\begin{aligned} \Omega _n \mapsto \tilde{\Omega }_n = \textrm{M}^{(1)^{-1}}\dots \textrm{M}^{(d)^{-1}} \Omega _n \textrm{M}^{(d)}\dots \textrm{M}^{(1)}\ . \end{aligned}$$

To evaluate these objects, we observe that

57 $$\begin{aligned} \textrm{M}^{(k)^{-1}} \gamma _n \textrm{M}^{(k)} =\frac{\gamma _n+ib_k\tilde{\gamma }_{n-k}}{\sqrt{1-b_k^2}} \quad \quad \quad \textrm{M}^{(k)^{-1}} \tilde{\gamma }_n \textrm{M}^{(k)} = \frac{\tilde{\gamma }_n-ib_k\gamma _{n+k}}{\sqrt{1-b_k^2}}\ , \end{aligned}$$

which, provided all $|b_k| \ne 1$, gives

$$\begin{aligned} \textrm{M}^{(k)^{-1}}\left( \sum _{\alpha =-m}^d s_\alpha \left( \gamma _{n+\alpha }\pm i \tilde{\gamma }_{n-\alpha }\right) \right) \textrm{M}^{(k)}&\propto \sum _{\alpha =-m}^d s_\alpha \left( \gamma _{n+\alpha }\pm i \tilde{\gamma }_{n-\alpha }\right) \\&\pm b_k \sum _{\alpha = k-d}^{k+m} s_{k-\alpha } \left( \gamma _{n+\alpha }\pm i \tilde{\gamma }_{n-\alpha }\right) \ . \end{aligned}$$

Define Algorithm 1 [19] for the coefficients $\{b_k\}$. This is designed such that each layer of the algorithm acting on $\Gamma _n$ eliminates the term $\left( \gamma _{n+\alpha }-i \tilde{\gamma }_{n-\alpha }\right)$ with the largest $\alpha$. As mentioned, this coincides with the Schur-Cohn algorithm [72, 73].

**Algorithm 1:**
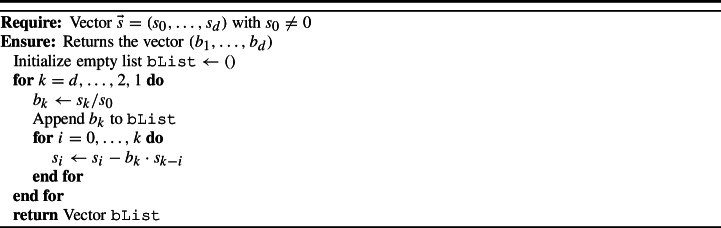
Compute $\{b_k\}$ from vector $\vec {s} = (s_0, \dots , s_d)$

To demonstrate this, note that

58 $$\begin{aligned} \begin{aligned} \textrm{M}^{(d)^{-1}}{\Gamma }_n \textrm{M}^{(d)}&\propto \sum _{\alpha =0}^d (s_\alpha - b_d s_{d-\alpha })\left( \gamma _{n+\alpha }-i \tilde{\gamma }_{n-\alpha }\right) \\&\propto \sum _{\alpha =0}^{d-1} s_\alpha ^{(d)}\left( \gamma _{n+\alpha }-i \tilde{\gamma }_{n-\alpha }\right) \ , \end{aligned} \end{aligned}$$

where $s_\alpha ^{(d)} = s_\alpha - b_d s_{d-\alpha }$. This leads to the recursion

59 $$\begin{aligned} \textrm{M}^{(k)^{-1}} \dots \textrm{M}^{(d)^{-1}}{\Gamma }_n \textrm{M}^{(d)}\dots \textrm{M}^{(k)} \propto \sum _{\alpha =0}^{k-1} s_\alpha ^{(k)}\left( \gamma _{n+\alpha }-i \tilde{\gamma }_{n-\alpha }\right) \ , \end{aligned}$$

where  for initial condition $s_\alpha ^{(d+1)} = s_\alpha$. After all *d* layers have been applied, this collapses onto $\tilde{\Gamma }_n \propto \gamma _{n}-i \tilde{\gamma }_{n}$. No terms, however, are necessarily eliminated in $\Omega _n$, and each layer instead generates a new term $\gamma _{n+\alpha }+i \tilde{\gamma }_{n-\alpha }$ with $\alpha = k-d$.

Therefore, for $N=2$, the transformation leads to

60 $$\begin{aligned} \begin{aligned} \tilde{H}_A&= \frac{1}{2}\sum _{n=1}^L\left( \sum _{\alpha =1-d}^d r'_\alpha \left( \gamma _{n}-i \tilde{\gamma }_{n}\right) \left( \gamma _{n+\alpha }+i \tilde{\gamma }_{n-\alpha }\right) - 2|\vec {s}|^2\right) \\&= \sum _{\alpha =1-d}^d r_\alpha (A_\alpha + G_\alpha ) + L(r_0-|\vec {s}|^2) \end{aligned} \end{aligned}$$

for some constants $\{r_\alpha (\vec {s})\}$. These are real functions of $\vec {s}$. While we do not need the explicit form of these coefficients, the computation can be done algorithmically. Algorithm 2 in Appendix C gives these coefficients up to an overall rescaling.

We may wonder if there has been any cancellation of coefficients in this representation that would give a non-vanishing contribution in general. However, for any linear dependence (aside from $G_k = G_{-k}$, which is true for all representations) to arise, we would need the transformation Eq. (54) to produce generators $A_l, G_m$ with *l*, *m* of order *L*. However, Eq. (60) is true for all *L*. If we consider $L \gg d,p$, then Eq. (54) cannot produce sufficiently large *l*, *m*. Therefore, $r_\alpha = a_\alpha = g_\alpha$ is the only possible set of *L*-independent coefficients generated by Eq. (54) for $N=2$. Moreover, Eq. (49) contains no constant term, and no linear combination of $A_l, G_m$ can give rise to this. Consequently, Algorithm 2 must give $r_0 = |\vec {s}|^2$, and

61 $$\begin{aligned} \tilde{H}_A=\sum _{\alpha =1-d}^d r_\alpha (A_\alpha + G_\alpha ) \end{aligned}$$

holds for all *N*. Note that the $\{r_\alpha (\vec {s})\}$ are independent of *N* and *L*.

As noted in Section 2.1, $|\psi _0\rangle$ is an eigenstate of $(A_\alpha +G_\alpha )$. Thus, it is an eigenstate of $\tilde{H}_A$. Reversing the transformation Eq. (54), we see that $|\varphi \rangle = \textrm{M}^{(d)}\dots \textrm{M}^{(1)}|\psi _0\rangle$ must therefore be an eigenstate of $H_A$, provided that $|b_k| \ne 1$ for all *k*. Following a similar procedure to that presented in Ref. [19], this can be expressed in MPS form: each operator $\textrm{M}^{(k)}$ is a local operator that can be expressed as an MPO with finite bond dimension, and $|\psi _0\rangle$ is a product state. Acting with a series of finite bond dimension MPOs on a product state gives an exact MPS.

Thus far, we have restricted ourselves to Hamiltonians with $f(z) = g(z)^2$. However, the Hamiltonians $f(z) = -g(z)^2$ take each $A_k \mapsto -A_k$. If $\{A_l, G_m|m,l\in \mathbb {Z}\}$ obey the Onsager algebra, then the generators $\{-A_l, G_m|m,l\in \mathbb {Z}\}$ obey the same structure. Therefore, to obtain the ground state of $f(z) = -g(z)^2$, we merely replace each $A_k\mapsto -A_k$ in every $\mathrm {M^{(k)}}$, and replace the ground state $|\psi _p\rangle$ of $A_p$ with the ground state $|\psi _p^-\rangle$ of $-A_p$.

Generalising this proof to any even $p=2k$ is trivial: the mapping $A_k \mapsto A_{k+1}, G_k \mapsto G_k$ preserves the structure of the Onsager algebra and Eq. (12) ensures that the ground state $|\psi _{2k}\rangle$ is unique. However, extending to any odd $p=2k+1$ requires more care, as Kramers-Wannier duality tells us that the ground state of $A_1$ is *N*-fold degenerate. Nevertheless, Result 1 holds for all *p*; $A_\alpha +G_\alpha$ commutes with *r*, and is therefore diagonalised for all $\alpha$ by the *N* eigenstates of *r* with distinct charges $0\le Q\le N-1$. We give a more detailed discussion in Appendix D.

### Proof of Result 2

We have established that, provided $|b_k| \ne 1$ for all *k*, the state $|\varphi \rangle$ given in Eq. (19) is an eigenstate of the Hamiltonian $H_A$ with $f(z)=\pm z^p g(z)^2$. Results of perturbation theory in finite-dimensional Hilbert spaces from Ref. [82] demonstrate that, provided the gap does not close, the ground state is an analytic function of $\vec {s}$. We apply this below to show that $|\varphi (\vec {s})\rangle$ is the ground state provided $H_A\in \mathcal {S}$.

#### The Ground State in the Gapped Region Containing $A_0$

We begin by noting that $|\varphi (\vec {S})\rangle = |\psi _0\rangle$ and $H_A(\vec {S}) = A_0$ for the vector $\vec {S} = (1,0,0,\dots )$. Thus, $|\varphi (\vec {s})\rangle$ is the ground state of $H_A(\vec {s})$ at $\vec {s} = \vec {S}$. Provided that the gap does not close, the ground state must be an analytic function of $\vec {s}$. Therefore, since $|\varphi (\vec {s})\rangle$ is an analytic function of $\vec {s}$ containing the ground state at $\vec {s}=\vec {S}$, it is the ground state everywhere in the gapped region surrounding $A_0$.

Note that when the gap closes, we find degeneracies, and this argument breaks down. We have therefore proved only that $|\varphi (\vec {s})\rangle$ is the ground state of $H_A(\vec {s})$ in the gapped region containing $A_0$.

#### The Ground State in the Gapped Region Containing $A_l$

The gapped region containing $A_0$ has every $|b_k| < 1$. If we instead have some $|b_k| > 1$, then we can use the identity

62 $$\begin{aligned} \text {arctanh}(b_k) = \text {arctanh}\left( \frac{1}{b_k}\right) + \frac{i \pi }{2} \end{aligned}$$

to write

63 $$\begin{aligned} \textrm{M}^{(k)} = \exp \left( -\beta _kA_k\right) = \exp \left( -\eta _kA_k\right) U_k\ , \end{aligned}$$

where we have defined

64 $$\begin{aligned} \eta _k = 2\text { arctanh}\left( \frac{1}{b_k}\right) \quad \quad \quad U_k = \exp \left( -i\pi A_k\right) \ . \end{aligned}$$

We can use the relation $U_k e^{-\beta _q A_q} = e^{-\beta _q A_{2k-q}}U_k$ from Eq. (11) to move all the unitary operators $U_k$ so that they act on the initial state $|\psi _0\rangle$. As shown in Ref. [31], this gives the ground state $|\psi _l\rangle$ of another fixed-point Hamiltonian $A_l$. Therefore, we are always able to write

65 $$\begin{aligned} |\varphi \rangle = \tilde{\textrm{M}}_{n_d}^{(d)}\dots \tilde{\textrm{M}}_{n_1}^{(1)} |\psi _l\rangle \ , \end{aligned}$$

where $$l \in \mathbb {Z}$$ and

66 $$\begin{aligned} \tilde{\textrm{M}}_{n}^{(k)} = \exp \left( -2\text { arctanh}(\tilde{b}_k) A_{n}\right) \end{aligned}$$

for $|\tilde{b}_k|<1$ and some integer *n*.

Although the state $|\varphi (\vec {s})\rangle$ is not smoothly connected to $|\psi _0\rangle$ along a path of gapped Hamiltonians $H_A$ if any of the $|b_k|>1$, it is always smoothly connected to a ground state $|\psi _l\rangle$ of a Hamiltonian $A_l$ for some $l \in \mathbb {Z}$. At this point, $H_A(\vec {s}) = A_l$. Therefore, the eigenstate $|\varphi (\vec {s})\rangle$ is the ground state provided $H_A \in \mathcal {S}$.

### Proof of Result 3

We can write the Laurent polynomial Eq. (14) describing a general Hamiltonian $H_A$ in the family Eq. (13) as

67 $$\begin{aligned} \begin{aligned} f(z)&= \sigma z^{P_0} \prod _{j=1}^{P_z} (z-z_j)\prod _{k=1}^{P_\zeta }(z-\zeta _k)\\&= \sum _{r=0}^{P_z+P_\zeta } f_r z^{r+P_0}\ , \end{aligned} \end{aligned}$$

where $\sigma \in \mathbb {R}$, $P_0 \in \mathbb {Z}$, $\{P_\zeta , P_z\}$ are non-negative integers, $|z_j|<1$, and $|\zeta _k|>1$. Note that this polynomial has no zeros on the unit circle. Such zeros correspond to a gap closing in $\mathcal {S}$ and, as discussed in Appendix E, the existence of zeros on the unit circle will give a $|b_k|=1$, which we exclude. If we now define

68 $$\begin{aligned} g(z) = \prod _{j=1}^{P_z} \sqrt{1-\frac{z_j}{z}}\prod _{k=1}^{P_\zeta }\sqrt{1-\frac{z}{\zeta _k}}\ , \end{aligned}$$

then, for $q = P_0 +P_z$, we have $f(z) =\pm z^q g(z)^2$. As overall scale is unimportant, we normalise the constant coefficient to $\pm 1$.

This polynomial *g*(*z*) can be expanded as a Laurent series

69 $$\begin{aligned} g(z) = \sum _{n=-\infty }^\infty s_n z^n\ , \end{aligned}$$

which is well-defined in the annulus $\mathscr {A}$ given by

70 $$\begin{aligned} \text {max}_j\{|z_j|\}< |z| < \text {min}_k\{|\zeta _k|\}\ . \end{aligned}$$

Now, truncating this series, we obtain

71 $$\begin{aligned} g_D(z) = \sum _{n=-D}^D s_n z^n\ , \end{aligned}$$

and

72 $$\begin{aligned} \begin{aligned} f_D(z) = \pm \sum _{n=-D}^D \sum _{m=-D}^D s_n s_m z^{n+m+q}=\pm \sum _{r=-2D}^{2D} c_r z^{r+q}\ , \end{aligned} \end{aligned}$$

which defines the Hamiltonian $H_A^{(D)}$. From this, we can define the new Hamiltonian  with Laurent polynomial

73 $$\begin{aligned} \delta f_D(z) = \pm \sum _{r=-2D}^{2D} (c_r-f_{r+P_z})z^{r+q}\ . \end{aligned}$$

In any sector, consider the ground states $|\varphi _D\rangle$ and $|\varphi \rangle$ of $H_A^{(D)}$ and $H_A$, respectively. Define the energy densities

74 $$\begin{aligned} \mathcal {E}_A = \frac{1}{L}\langle \varphi |H_A|\varphi \rangle \quad \quad \quad \quad \mathcal {E}_A^{(D)} = \frac{1}{L}\langle \varphi _D|H_A|\varphi _D\rangle . \end{aligned}$$

From variational principles, we have75By definition of the spectral norm, for any normalised state $|\psi \rangle$, we have $|\langle \psi |\delta H_A^{(D)}|\psi \rangle | \le ||\delta H_A^{(D)}||$. Therefore, using the triangle inequality, the energy densities differ by76where $\mathscr {E}_{\text {max}}$ is a bound on the energy density of the Onsager generators $A_l$ (assumed in general to be *O*(1)). In the chiral clock model, $\mathscr {E}_{\text {max}} = (N-1)/2N$ [31].

The coefficients in Eq. (73) can be evaluated using residue calculus. This allows us to bound the magnitude of each term as follows:

77 $$\begin{aligned} \begin{aligned} |c_r-f_{r+P_z}|&= \left| \frac{1}{2\pi i} \oint _{|z|=\rho } \frac{\delta f_D(z)}{z^{r+q+1}}\,\textrm{d}z \right| \\&\le \frac{M_\rho (D)}{\rho ^{r+q}}\ , \end{aligned} \end{aligned}$$

where $$\text {max}_j\{|z_j|\}< \rho < \text {min}_k\{|\zeta _k|\}$$ and

78 $$\begin{aligned} M_\rho (D) = \sup _{|z|=\rho } |\delta f_D(z)|\ . \end{aligned}$$

Choosing $\text {max}_j\{|z_j|\}< \rho _-<1<\rho _+ < \text {min}_k\{|\zeta _k|\}$, we can write

79 $$\begin{aligned} \begin{aligned} \sum _{r=-2D}^{2D} |c_r-f_{r+P_z}|&\le M_{\rho _+}(D) \sum _{r=-q}^{2D} \frac{1}{\rho _+^{r+q}} + M_{\rho _-}(D) \sum _{r=-2D}^{-1-q} \frac{1}{\rho _-^{r+q}}\\&\le \text {max}\{M_{\rho _+}(D), M_{\rho _-}(D)\}\Sigma \ , \end{aligned} \end{aligned}$$

where we have defined the finite

80 $$\begin{aligned} \Sigma = \left( \frac{\rho _+}{\rho _+ -1}+\frac{\rho _-}{1-\rho _-}\right) \ . \end{aligned}$$

However, convergence of $f_D(z)$ to *f*(*z*) in the annulus $\mathscr {A}$ implies that for any $\epsilon >0$ we can fix a sufficiently large *D* (and in the background an $L\gg D$) such that $M_{\rho _\pm }(D) < \epsilon$, giving

81 $$\begin{aligned} \sum _{r=-2D}^{2D} |c_r-f_{r+P_z}|\le \epsilon \Sigma \ , \end{aligned}$$

and therefore that82Hence, the MPS skeleton is dense: for any Hamiltonian $H_A\in \mathcal {S}$ in the Onsager-integrable chiral clock class, we can construct a Hamiltonian $H_A^{(D)}$ on the skeleton with a ground state of the form

83 $$\begin{aligned} |\varphi _D\rangle = \textrm{M}^{(d)}\dots \textrm{M}^{(1)} |\psi _p^\pm \rangle \ , \end{aligned}$$

with an energy density with respect to $H_A$ approaching the ground-state energy density of $H_A$. Thus, we have a method for approximating the ground state of any Hamiltonian in $\mathcal {S}$. Moreover, by the same reasoning, the ground state of $H_A^{(D)}$ in any particular sector approximates the ground state of $H_A$ in that sector for sufficiently large *D*.

### Proof of Result 4

As shown in Section 5.1, we can write

84 $$\begin{aligned} \tilde{H}_A = \sum _{\alpha =1-d}^dr_\alpha (A_\alpha +G_\alpha )\ . \end{aligned}$$

As noted in Section 2.1, the states $|\phi ^{(P)}_{E_0+1/N,\,p=0}\rangle$ are eigenstates of $(A_\alpha +G_\alpha )$. Thus, these states are eigenstates of $\tilde{H}_A$. Reversing the transformation Eq. (54), we see that $|\chi _{p=0}^{(P)}\rangle = \textrm{M}^{(d)}\dots \textrm{M}^{(1)}|\phi ^{(P)}_{E_0+1/N,\, p=0}\rangle$ are eigenstates of $H_A$, provided that $|b_k| \ne 1$ for all *k*.

Similarly to the ground state, $|\phi ^{(P)}_{E_0+1/N,\, p=0}\rangle$ has an exact MPS representation. In analogy with the *W*-state [6], the most efficient MPS representation is

85 $$\begin{aligned} \begin{aligned} |\phi ^{(P)}_{E_0+1/N,\, p=0}\rangle&= \frac{1}{\sqrt{L}}\sum _{j=1}^L e^{-iPj}Z_j^\dagger |\psi _0\rangle \\&= \sum _{\{\sigma _j \in \{0,1\}\}} \langle L| \mathcal {A}_1^{(\sigma _1)} \dots \mathcal {A}_L^{(\sigma _L)}|R\rangle |\sigma _1 \dots \sigma _L\rangle \ , \end{aligned} \end{aligned}$$

where we have defined the vectors

86 $$\begin{aligned} |L\rangle = \begin{pmatrix}1\\ 0\end{pmatrix} \quad \quad \quad |R\rangle = \begin{pmatrix}0\\ 1\end{pmatrix} \end{aligned}$$

and the site-dependent matrices

87 $$\begin{aligned} \mathcal {A}_j^{(0)} = \begin{pmatrix}1 &  0\\ 0 &  1\end{pmatrix} \quad \quad \quad \mathcal {A}_j^{(1)} = \frac{1}{\sqrt{L}}\begin{pmatrix}0 &  e^{- i P j}\\ 0 &  0\end{pmatrix}\ . \end{aligned}$$

As discussed in Section 5.1, each $\textrm{M}^{(k)}$ can be expressed as an MPO with finite bond dimension. Applying a series of such MPOs to an exact MPS yields another MPS of finite bond dimension. Thus, the eigenstate $|\chi _{p=0}^{(P)}\rangle$ has a representation as an exact MPS.

Moreover, for $p\ne 0$, keeping *p* even, we can use Eq. (12) to obtain eigenstates

88 $$\begin{aligned} |\chi _{p}^{(P)}\rangle = \textrm{M}^{(d)}\dots \textrm{M}^{(1)}|\phi ^{(P)}_{E_0+1/N,\, p}\rangle \end{aligned}$$

as defined in Eq. (21). Note that the pivot procedure defined in Ref. [31] ensures that $|\phi ^{(P)}_{E_0+1/N,\, p}\rangle$ also has an exact MPS representation. Unlike in the ground state case, it is unclear whether this could be generalised for odd *p*, as Kramers-Wannier dual Hamiltonians with periodic boundary conditions cannot have a single domain wall.

### Constraints on Coefficients $\{r_\alpha \}$

In this section, we identify some constraints on the coefficients $r_\alpha$ appearing above. We have

89 $$\begin{aligned} \tilde{H}_A |\psi _0\rangle = \varepsilon _\varphi |\psi _0\rangle \quad \implies \quad H_A|\varphi \rangle = \varepsilon _\varphi |\varphi \rangle . \end{aligned}$$

Hence, the energy of the eigenstate $|\varphi \rangle$ of the Hamiltonian $H_A$ with $f(z)=g(z)^2$ is given by

90 $$\begin{aligned} \begin{aligned} \varepsilon _\varphi&= \langle \varphi |H_A|\varphi \rangle = \langle \psi _0|\tilde{H}_A|\psi _0\rangle \\&= \sum _{\alpha =1-d}^d r_\alpha \langle \psi _0|A_\alpha |\psi _0\rangle \\&= r_0 \langle \psi _0|A_0|\psi _0\rangle +\sum _{\alpha =1}^d (r_\alpha +r_{-\alpha })\langle \psi _0|A_\alpha |\psi _0\rangle \ , \end{aligned} \end{aligned}$$

as the ladder operators Eq. (7) impose that $\langle \psi |A_\alpha |\psi \rangle = \langle \psi |A_{-\alpha }|\psi \rangle$ for any eigenstate $|\psi \rangle$ of $A_0$.

Defining $E^\pm _k = J_x^k \pm i J_y^k$, it follows from Eq. (42) that

91 $$\begin{aligned} \langle \psi _0|A_\alpha |\psi _0\rangle = \sum _{k=1}^n \cos (m\theta _k) \langle \psi _0|J_k^x|\psi _0\rangle = -S \sum _{k=1}^n \cos (m\theta _k) \end{aligned}$$

for spin *S*. For these Hamiltonians $H_A$, we can use this and Eq. (46) to write the ground state energy as

92 $$\begin{aligned} \begin{aligned} \varepsilon _0 = -S \sum _{l,m=0}^d s_l s_m \sum _{k=1}^n \cos ((l-m)\theta _k) = \sum _{l,m=0}^d s_l s_m \langle \psi _0|A_{l-m}|\psi _0\rangle . \end{aligned} \end{aligned}$$

We know that $|\varphi \rangle$ is the ground state of the “ground-state Onsager sector” for Hamiltonians $H_A$. Thus, in this region, $\varepsilon _\varphi = \varepsilon _0$. From this, we can conclude that $r_0 = |\vec {s}|^2$ (as we had already argued) and that

93 $$\begin{aligned} r_\alpha + r_{-\alpha } = 2\sum _{\begin{array}{c} l,m=0\\ l-m=\alpha \end{array}}^d s_l s_m \quad \quad \quad \alpha \in \{1,2,\dots ,d\}\ . \end{aligned}$$

Algorithm 2 will therefore give coefficients satisfying this relationship.

## Outlook

In this work, we uncovered dense MPS skeletons in general classes of Onsager-integrable Hamiltonians, each analogous to that in Ref. [19]. While certain results hold for any representation, we deal primarily with the Onsager-integrable chiral clock chains—these are the key physical representations of this class. Due to the interacting nature of these models, any analysis is more involved than in the free-fermion classes considered previously. Nevertheless, we constructed the ground state explicitly for chiral clock Hamiltonians lying on the skeleton in gapped regions of the phase diagram containing any fixed-point Hamiltonian $A_l$. Moreover, we showed that the constructed state remains an eigenstate everywhere on the skeleton, excluding a measure-zero set of cases. Given that the skeleton is dense, we also have a method for approximating the ground state of any model in these gapped regions as the limit of a sequence of states of the form Eq. (19).

To prove the existence of the MPS skeleton, we worked in a specific representation of the Onsager algebra, considering Hamiltonians of the form $H_A = \pm \sum _{k,q} s_k s_q A_{k+q+p}$. We emphasise that, even fixing $N=2$, the method used differs from the proof presented in Ref. [19]. However, it is not fully independent, since we utilise features of the frustration-free form of the Hamiltonian derived in this work. We note that the methods used in earlier work on the BDI skeleton rely heavily on the free-fermion representation. It would be interesting to explore whether our models are frustration-free for $N>2$, potentially using the parafermionic representation, and to understand whether the Witten conjugation method can be used in this context [83]. Note that any such application will be complicated by the level crossing transitions at the boundary of $\mathcal {S}$.

We calculated the ground state expectation of the disorder operator for the $d=1$ leg of the skeleton, showing how the well-understood $N=2$ formula generalises to all even *N*. Whether the same generalisation occurs for larger *d* remains an interesting question that we plan to explore in future work. Our derivation of the disorder operator on the skeleton gives a new route to understanding correlations in this family of models. Away from the superintegrable chiral Potts line, a formula for the disorder parameter is not known, despite the analogous result being established for the $N=2$ case using Toeplitz determinant theory [47]. Baxter gives a determinantal formula for this operator on the superintegrable chiral Potts line [84]. We expect that it will be possible to construct similar determinantal results away from this line using the MPS skeleton: if we establish a closed formula on the skeleton, we could use the density of the skeleton to obtain a general formula.

Importantly, we contrast our results with existing literature on MPS in Bethe ansatz solvable models, as Bethe ansatz eigenstates can be placed in an MPS framework [85–89]. While this gives an MPS form for all eigenstates, the corresponding bond dimension can diverge in the thermodynamic limit (dependent on the magnetisation of the state), and naturally relies on a *U*(1) symmetry labelling this magnetisation. In $H_A$, the charge is conserved modulo *N*. Thus, we cannot apply these results to find exact MPS ground states of our family of models. It would be interesting to see if we could use our exact ground states to feed into a further Bethe ansatz analysis, or to connect to the eigenvector constructions of Refs. [43, 44]. Our MPS skeleton states could also be useful initial states for numerics.

Our MPS construction gives an upper bound on the number of non-zero entanglement eigenvalues of the ground state. In Refs. [19, 20], this construction was conjectured to be optimal for $N=2$, which could be proved in certain cases. It is unclear whether this optimality also applies to the clock models.

In Ref. [90], quantum scar states are constructed via imaginary time evolutions with certain Onsager algebra elements. These are MPS eigenstates in a non-integrable variant of the model in Ref. [33]. It would be interesting to understand if our construction would be useful in this context.

Finally, we constructed a further set of *L* exact MPS eigenstates. These are excited states in the aforementioned gapped regions of the phase diagram. We could use these states to place partial constraints on the location of the phase boundaries: the gap will certainly close when any one of these states becomes degenerate with $|\varphi \rangle$. However, this would not determine the phase boundaries entirely. Therefore, we leave the characterisation of the phase boundaries for further work.

## Figures and Tables

**Algorithm 2:**
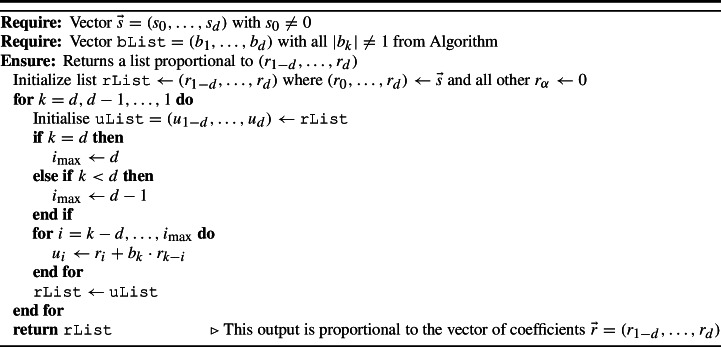
Compute coefficients $\{r_\alpha \}$ from vector $\vec {s} = (s_0, \dots , s_d)$

## Data Availability

No data were created or analysed in this study.

## Properties of $(A_n+G_n)$

To prove Eq. (16), we note that the Onsager algebra admits the ladder operators  and $R_{m,l}^\dagger$ given by [69]

94 $$\begin{aligned} R_{m,l} = \frac{1}{2}\big [[A_m,A_l], A_l\big ] + \frac{1}{2}[A_m, A_l] = \frac{1}{4}A_m + \frac{1}{2}G_{m-l}-\frac{1}{4}A_{2l-m}\ . \end{aligned}$$

Given an eigenstate $|\psi \rangle$ of $A_l$ with energy *E*, these obey

95 $$\begin{aligned} A_l (R_{m,l}|\psi \rangle ) = (E -1)(R_{m,l}|\psi \rangle ) \quad \quad \quad A_l (R^\dagger _{m,l}|\psi \rangle ) = (E +1)(R^\dagger _{m,l}|\psi \rangle )\ . \end{aligned}$$

It follows that

96 $$\begin{aligned} \begin{aligned} A_0 (A_n+G_n)|\psi _0\rangle&=(A_n+G_n)A_0|\psi _0\rangle + [A_0, A_n+G_n] |\psi _0\rangle \\&= E_0 (A_n+G_n)|\psi _0\rangle -2 R_{n, 0} |\psi _0\rangle \ . \end{aligned} \end{aligned}$$

However, since $|\psi _0\rangle$ is the ground state of $A_0$, there exists no eigenstate with energy $E_0-1$. Therefore, $R_{n,0} |\psi _0\rangle = 0$, and we have

97 $$\begin{aligned} A_0 (A_n+G_n)|\psi _0\rangle = E_0 (A_n+G_n)|\psi _0\rangle \ . \end{aligned}$$

Thus, acting with $(A_n+G_n)$ on $|\psi _0\rangle$ keeps us within the ground state sector of $A_0$. Given that this ground state is unique, we must have

98 $$\begin{aligned} (A_n+G_n)|\psi _0\rangle \propto |\psi _0\rangle \ . \end{aligned}$$

Additionally, any eigenstate $|\psi \rangle$ of $A_0$ with energy *E* that is annihilated by $R_{n,0}$ obeys

99 $$\begin{aligned} A_0 (A_n+G_n)|\psi \rangle = E (A_n+G_n)|\psi \rangle \ . \end{aligned}$$

Thus, $(A_n+G_n)|\psi \rangle$ acting on $|\psi \rangle$ keeps us within the eigenspace of $A_0$ with energy *E*. Hence, if all states within this eigenspace obey $R_{n,0}|\psi \rangle = 0$, it is possible to find a basis $\{|\phi _E^{(i)}\rangle \}$ such that

100 $$\begin{aligned} (A_n+G_n)|\phi _E^{(i)}\rangle \propto |\phi _E^{(i)}\rangle \ . \end{aligned}$$

Given that the lowest charge *m* excitations have energy $E_0+m/N$ [31], and that $R_{n,0}|\phi _E\rangle \propto |\phi _{E-1}\rangle$, states with $m<N$ can be put in bases satisfying (100), as $R_{n,0}$ must annihilate all these states.

We note that each $(A_n+G_n)$ commutes with the translation operator. Thus, for single-particle excitations, for which there are *L* possible states, the appropriate basis of states $\{|\phi ^{(P)}_{E_0+1/N}\rangle \}$ that satisfy (100) is given by the *L* eigenstates of the translation operator

101 $$\begin{aligned} |\phi ^{(P)}_{E_0+1/N}\rangle = \frac{1}{\sqrt{L}}\sum _{j=1}^L e^{-i P j} Z_j^\dagger |\psi _0\rangle \quad \quad \quad P \in \bigg \{0, \frac{2\pi }{L}, \frac{4\pi }{L} , \dots , \frac{2(L-1)\pi }{L}\bigg \}\ . \end{aligned}$$

We therefore have

102 $$\begin{aligned} (A_n+G_n) |\phi ^{(P)}_{E_0+1/N}\rangle \propto |\phi ^{(P)}_{E_0+1/N}\rangle \ . \end{aligned}$$

## Disorder operator for $d=1$

For even *N*, the unitary disorder operator is given by

103 $$\begin{aligned} \mu _k = \prod _{j=-\infty }^k X_j^{N/2}\ . \end{aligned}$$

Choose $|a|<1$ so that $\beta \in \mathbb {R}$. We aim to evaluate

104 $$\begin{aligned} \langle \mu _k\rangle = \frac{\langle \varphi |\mu _k|\varphi \rangle }{\langle \varphi |\varphi \rangle } = \frac{\langle \psi _0|e^{-\beta A_1}\mu _ke^{-\beta A_1}|\psi _0\rangle }{\langle \psi _0|e^{-2\beta A_1}|\psi _0\rangle }\ . \end{aligned}$$

### Simplifying the Numerator

In this section, we simplify

105 $$\begin{aligned} \langle \varphi |\mu _k|\varphi \rangle = \langle \psi _0|e^{-\beta A_1} \mu _ke^{-\beta A_1} |\psi _0\rangle \ . \end{aligned}$$

Firstly, define

106

and

107 $$\begin{aligned} U_{j,j+1}^{(m)}(\beta ) = \exp \left( \frac{\beta }{N}\alpha _m(Z_{j}^\dagger Z_{j+1})^m\right) \ , \end{aligned}$$

so that

108 $$\begin{aligned} e^{-\beta A_1} = \prod _{j=1}^L \prod _{m=1}^{N-1} U_{j, j+1}^{(m)}(\beta )\ . \end{aligned}$$

Moreover, since $\mu _k$ is unitary, we have

109 $$\begin{aligned} \mu _k e^{-\beta A_1} \mu _k^\dagger = e^{-\beta \mu _k A_1 \mu _k^\dagger }\ . \end{aligned}$$

Given that $X_j Z_k = \omega ^{\delta _{jk}}Z_k X_j$, we have

110

Therefore,

111 $$\begin{aligned} \mu _k e^{-\beta A_1}= e^{-\beta \mu _k A_1 \mu _k^\dagger } \mu _k= e^{-\beta A_1} \prod _{\begin{array}{c} m=1 \\ m \text { odd} \end{array}}^{N-1} U^{(m)}_{k,k+1}(-2\beta ) \mu _k\ . \end{aligned}$$

Given that $\mu _k |\psi _0\rangle = |\psi _0\rangle$, we arrive at

112 $$\begin{aligned} \langle \psi _0|e^{-\beta A_1}\mu _ke^{-\beta A_1}|\psi _0\rangle =\langle \psi _0|e^{-2\beta A_1} \prod _{\begin{array}{c} m=1 \\ m \text { odd} \end{array}}^{N-1}U_{k,k+1}^{(m)}(-2\beta ) |\psi _0\rangle \ . \end{aligned}$$

### The Transfer Matrix Method

If we write $|\psi _0\rangle$ in terms of the states $|a\rangle$ that are diagonal in the *Z*-basis

113 $$\begin{aligned} |\psi _0\rangle = \frac{1}{\sqrt{N^L}}\sum _{a_1\dots a_L =0}^{N-1} |a_1 \dots a_L\rangle \ , \end{aligned}$$

then

114 $$\begin{aligned} {\langle \psi _0|e^{-2\beta A_1}|\psi _0\rangle } = \frac{1}{N^L}\sum _{a_1 \dots a_L=0}^{N-1}\prod _{j=1}^L\exp \left( \frac{2\beta }{N} F_1(a_{j-1}, a_j)\right) \end{aligned}$$

and

115 $$\begin{aligned} \langle \psi _0|e^{-2\beta A_1} \prod _{\begin{array}{c} m=1 \\ m \text { odd} \end{array}}^{N-1}U_{k,k+1}^{(m)}(-2\beta ) |\psi _0\rangle =&\frac{1}{N^L}\sum _{a_1 \dots a_L=0}^{N-1}\prod _{j=1}^L\exp \left( \frac{2\beta }{N} F_1(a_{j-1}, a_j)\right) \nonumber \\&\exp \left( -\frac{2\beta }{N} F_2(a_k, a_{k+1})\right) \ , \end{aligned}$$

where we have defined

116 $$\begin{aligned} F_1(x,y) = \sum _{m=1}^{N-1}\alpha _m \omega ^{-m(x-y)} = {\left\{ \begin{array}{ll} \frac{N-1}{2}-(x-y) &  0 \le x-y< N \\ \frac{-N-1}{2}-(x-y) &  -N \le x-y < 0 \end{array}\right. } \end{aligned}$$

117 $$\begin{aligned} F_2(x,y) = \sum _{\begin{array}{c} m=1 \\ m \text { odd} \end{array}}^{N-1}\alpha _m \omega ^{-m(x-y)} = {\left\{ \begin{array}{ll} N/4 &  \begin{array}{l} 0 \le x - y< N/2 \\ -N \le x - y< -N/2 \end{array} \\ -N/4 &  \begin{array}{l} -N/2 \le x - y< 0 \\ N/2 \le x - y < N\ . \end{array} \end{array}\right. } \end{aligned}$$

The simplification for $F_1(x,y)$ follows from a trigonometric identity, given as Eq. (27) in Ref. [31]. The simplification for $F_2(x,y)$ follows from writing

118 $$\begin{aligned} F_2(x,y) = F_1(x,y) - \sum _{\begin{array}{c} m=1 \\ m \text { even} \end{array}}^{N-1}\alpha _m \omega ^{-m(x-y)}\ . \end{aligned}$$

The second sum can be rearranged into a form analogous to $F_1(x,y)$, which can be similarly simplified with trigonometric identities.

If we define the matrices with entries

119 $$\begin{aligned} T_{a,b} = \exp \left( \frac{2\beta }{N} F_1(a,b)\right) \quad \quad \quad B_{a,b} = \exp \left( \frac{2\beta }{N} \left( F_1(a,b)-F_2(a,b)\right) \right) \ , \end{aligned}$$

then, using the cyclic property of the trace,

120 $$\begin{aligned} \langle \mu _k\rangle = \frac{\text {Tr}(T^{L-1} B)}{\text {Tr}(T^L)} \implies \lim _{L\rightarrow \infty }\langle \mu _k\rangle = \frac{\vec {v}_0^T B \vec {v}_0}{\lambda _0}\ , \end{aligned}$$

since, in the thermodynamic limit $L \rightarrow \infty$, the largest eigenvalue $\lambda _0$ of the transfer matrix *T*, with eigenvector $\vec {v}_0$, dominates.

For general even *N*, we find the circulant transfer matrix

121 $$\begin{aligned} T = \begin{pmatrix} e^{\beta (N-1)/N} &  e^{-\beta (N-1)/N} &  \cdots \\ e^{\beta (N-3)/N} &  e^{\beta (N-1)/N} &  \cdots \\ e^{\beta (N-5)/N} &  e^{\beta (N-3)/N} &  \cdots \\ \vdots &  \vdots &  \ddots &  \\ e^{-\beta (N-1)/N} &  e^{-\beta (N-3)/2N} &  \cdots \\ \end{pmatrix}. \end{aligned}$$

Each column in this matrix is a cyclic permutation of the first one, which has *N* entries $T_k$ defined by

122 $$\begin{aligned} T_k = e^{\beta (N-(2k+1))/N} \quad \quad \quad \text {for} \quad \quad \quad k \in \{0,1,\dots ,N-1\}\ . \end{aligned}$$

The matrix *B* is similarly circulant, with each column being a cyclic permutation of the initial column, having *N* entries $B_k$ defined by

123 $$\begin{aligned} B_k = {\left\{ \begin{array}{ll} e^{-\beta /2}T_k \quad & \text {for} \quad k \in \{0,1,\dots ,N/2-1\}\\ e^{\beta /2}T_k \quad & \text {for} \quad k \in \{N/2,\dots ,N-1\} \end{array}\right. }\ . \end{aligned}$$

A circulant matrix can be diagonalised using standard results [78]. By the triangle inequality, the largest eigenvalue of *T* (and *B*) corresponds to the eigenvector

124 $$\begin{aligned} \vec {v}_0 = \frac{1}{\sqrt{N}}(1,1,\dots ,1)\ . \end{aligned}$$

Thus, using $\beta =2 \,\text {arctanh}(a)$, we obtain

125 $$\begin{aligned} \begin{aligned} \lim _{L\rightarrow \infty }\langle \mu _k\rangle&= \frac{\sum _{k=0}^{N/2-1} \cosh \left( -\frac{\beta }{2N}(N-2(2k+1))\right) }{\sum _{k=0}^{N/2-1}\cosh \left( -\frac{\beta }{2N}(2N-2(2k+1))\right) }\\&= \frac{1}{\cosh (-\beta /2)}\\&= \sqrt{1-a^2} \end{aligned} \end{aligned}$$

for all even *N*.

## Algorithm for Finding the Coefficients $\{r_\alpha \}$

Should we wish to calculate the coefficients in Eq. (60), this can be done algorithmically as shown below in Algorithm 2. To demonstrate the operation of this algorithm, we use (58) to find

126 $$\begin{aligned} \begin{aligned} \textrm{M}^{(d)^{-1}}{\Omega }_n \textrm{M}^{(d)}&\propto \sum _{\alpha =0}^d s_\alpha \left( \gamma _{n+\alpha }+i \tilde{\gamma }_{n-\alpha }\right) + \sum _{\alpha =0}^d b_d s_{d-\alpha }\left( \gamma _{n+\alpha }+i \tilde{\gamma }_{n-\alpha }\right) \\&\propto \sum _{\alpha =0}^d r_\alpha ^{(d)}\left( \gamma _{n+\alpha }+i \tilde{\gamma }_{n-\alpha }\right) \ . \end{aligned} \end{aligned}$$

The next layer yields

127 $$\begin{aligned} \textrm{M}^{(d-1)^{-1}}\textrm{M}^{(d)^{-1}}{\Omega }_n \textrm{M}^{(d)}\textrm{M}^{(d-1)}&\propto \sum _{\alpha =0}^d r_\alpha ^{(d)}\left( \gamma _{n+\alpha }+i \tilde{\gamma }_{n-\alpha }\right) \nonumber \\&\quad +b_{d-1}\sum _{\alpha =-1}^{d-1}r_{d-1-\alpha }^{(d)}\left( \gamma _{n+\alpha }+i \tilde{\gamma }_{n-\alpha }\right) \nonumber \\&\propto \sum _{\alpha = -1}^d r_\alpha ^{(d-1)}\left( \gamma _{n+\alpha }+i \tilde{\gamma }_{n-\alpha }\right) \ , \end{aligned}$$

with the third layer giving

128 $$\begin{aligned} \begin{aligned} \textrm{M}^{(d-2)^{-1}}\textrm{M}^{(d-1)^{-1}}\textrm{M}^{(d)^{-1}}{\Omega }_n \textrm{M}^{(d)}\textrm{M}^{(d-1)}\textrm{M}^{(d-2)}&\propto \sum _{\alpha =-1}^d r_\alpha ^{(d-1)}\left( \gamma _{n+\alpha }+i \tilde{\gamma }_{n-\alpha }\right) \\&\quad +b_{d-2}\sum _{\alpha =-2}^{d-1}r_{d-2-\alpha }^{(d-1)}\left( \gamma _{n+\alpha }+i \tilde{\gamma }_{n-\alpha }\right) \\&\propto \sum _{\alpha = -2}^d r_\alpha ^{(d-2)}\left( \gamma _{n+\alpha }+i \tilde{\gamma }_{n-\alpha }\right) \ , \end{aligned} \end{aligned}$$

where we have defined $r_\alpha ^{(k)} = r_\alpha ^{(k+1)} + b_k r_{k-\alpha }^{(k+1)}$ and $r_\alpha ^{(d+1)} = s_\alpha$. Proceeding in this manner gives Algorithm 2. Note that the second sum initially has an upper limit of *d*, but for every following layer, it has an upper limit of $d-1$. This follows directly from Eq. (58).

## Understanding Odd *p*

### Handling Degeneracy

Consider generators $\{A_l, G_m|l,m\in \mathbb {Z}\}$ obeying the Onsager algebra. As noted in Section 2, making the replacements $A_k \rightarrow A_{k+1}, G_k \rightarrow G_k$ gives a new set of generators obeying the Onsager algebra. As our proof in Section 5.1 relies only on the structure of the Onsager algebra and the uniqueness of the ground state, given that

129 $$\begin{aligned} H_A^{p=0} = \sum _{k,q=0}^d s_k s_q A_{k+q} \end{aligned}$$

has an eigenstate $|\varphi ^{p=0}\rangle = e^{-\beta _d A_d}\dots e^{-\beta _1 A_1} |\psi _0\rangle$, applying the above mapping between the generators *p* times, we can conclude that

130 $$\begin{aligned} H_A^{p} = \sum _{k,q=0}^d s_k s_q A_{k+q+p} \end{aligned}$$

has an eigenstate

131 $$\begin{aligned} |\varphi ^{p}\rangle = e^{-\beta _d A_{d+p}}\dots e^{-\beta _1 A_{1+p}} |\psi _{p}\rangle . \end{aligned}$$

This is of the form given in Result 1. However, we have ignored a subtlety: for $|\psi _0\rangle$ to be an eigenstate of each $(A_{\alpha } + G_\alpha )$, we used the fact that the ground state is unique. This is discussed in Appendix A. As $A_{2k}$ is related to $A_{0}$ by the unitary transformation (12), the ground state is also unique for even *p*, and the argument above holds.

However, for clock models, $A_1$ instead has a set of *N* degenerate ground states $\{|\psi _1^{(n)}\rangle \}$; any $A_{2k+1}$, being unitarily related to $A_1$, will similarly have ground-state degeneracy *N*. Therefore, our argument breaks down unless we can find a basis of $\{|\psi _1^{(n)}\rangle \}$ that simultaneously diagonalises every $(A_{\alpha +1}+G_\alpha )$, as we now have (following the argument in Appendix A)

132 $$\begin{aligned} (A_{\alpha +1} + G_\alpha )\{|\psi _1^{(n)}\rangle \} = \sum _m c_m \{|\psi _1^{(m)}\rangle \} \end{aligned}$$

for some constants $\{c_m\}$. However, as noted in Section 4, the operator $r= \prod _{j=1}^L X_j$

commutes with every generator $A_l, G_m$. Thus, for all $\alpha$, the appropriate basis diagonalising $(A_{\alpha +1}+G_\alpha )$ must also diagonalise *r*.

Working in the *Z*-diagonal basis, $A_1$ has *N* degenerate ground states $|\tau _a\rangle = |aaa\dots \rangle$ for $a \in \{0,1,\dots ,N-1\}$. The unique basis that diagonalises *r* is

133 $$\begin{aligned} |\psi _1^{(n)}\rangle = \sum _{a=0}^{N-1} \omega ^{-na} |\tau _a\rangle \quad \quad \quad n\in \{0,1,\dots ,N-1\}\ . \end{aligned}$$

Each of these states has charge $Q=n$. As *r* commutes with all $A_l, G_m$, the basis $\{|\psi _1^{(n)}\rangle \}$ with distinct charges must diagonalise $(A_{\alpha +1}+G_\alpha )$ for all $\alpha$. Thus, despite the fact that $A_1$ does not have a unique ground state, Result 1 still holds. The pivot procedure (12) allows us to draw the same conclusion for all odd *p*.

### A Note on Chain Length

If we wish to find the ground state of a Hamiltonian with $f(z) = -z^p g(z)^2$, we can perform a similar analysis by replacing $A_k \rightarrow -A_k$ and $|\psi _p\rangle \rightarrow |\psi _p^-\rangle$. For odd *p*, we again encounter the obstacle of degeneracy. For $-A_1$, if $L = 0 \mod N$, we have *N* degenerate ground states of the form $|\upsilon \rangle = T^{\upsilon }|0,1,2,\dots ,N-1,0,1,2,\dots \rangle$, where *T* is the translation operator and $\upsilon \in \{0,1,\dots ,N-1\}$. As before, we can put these states into a basis that simultaneously diagonalises every $A_{\alpha +1}+G_\alpha$:

134 $$\begin{aligned} |\psi _1^{(n),-}\rangle = \sum _{\upsilon =0}^{N-1} \omega ^{n\upsilon }|\upsilon \rangle \ . \end{aligned}$$

However, if we do not have $L = 0 \mod N$, we obtain frustrated ground states, making a similar analysis more difficult. We choose $L=0\mod N$ to avoid this complication.

## Zeros on the Unit Circle

In this appendix, we make the connection between zeros of *g*(*z*) on the unit circle, and having some $|b_k|=1$ appearing when we apply Algorithm 1.

We begin with the polynomial *g*(*z*) of degree *d* and its reciprocal polynomial $\tilde{g}(z) = z^d g(1/z)$ given by

135 $$\begin{aligned} g(z) = \sum _{k=0}^d s_k z^k \quad \quad \quad \tilde{g}(z) = \sum _{k=0}^d s_{d-k}z^k\ , \end{aligned}$$

where the coefficients $s_k$ are real. Assume further that $s_0, s_d\ne 0$. It follows that *g*(*z*) and $\tilde{g}(z)$ must share any zeros on the unit circle (along with their multiplicities).

Now define the Schur transform, which is a polynomial of the form

136 $$\begin{aligned} \begin{aligned} Tg(z)&= g(z) - \frac{\tilde{g}(0)}{g(0)}\tilde{g}(z)\\&= \sum _{k=0}^d \left( s_k - \frac{s_d}{s_0} s_{d-k}\right) z^k\\&= \sum _{k=0}^{d-1} s^{(d)}_k z^k, \end{aligned} \end{aligned}$$

for $s^{(d)} = (s_k - s_d s_{d-k}/s_0)$. Since $s^{(d)}_d=0$, *Tg*(*z*) is a polynomial of lower degree than $g, \tilde{g}$. Defining $T^0g(z) = g(z)$, we can iteratively construct the series of polynomials for $0\le n\le d$

137 $$\begin{aligned} T^n g(z) = T \left( T^{n-1}g(z)\right) = \sum _{k=0}^{d-n}s_k^{(d-(n-1))}z^k\ , \end{aligned}$$

where

138 $$\begin{aligned} s_k^{(m)} = s_k^{(m+1)} - \frac{s_{m}^{(m+1)}}{s_0^{(m+1)}} s_{m-k}^{(m+1)}\quad \quad \quad \text {and}\quad \quad \quad s^{(d+1)}_k = s_k\ . \end{aligned}$$

Moreover, Algorithm 1 defines

139 $$\begin{aligned} b_m = \frac{s_m^{(m+1)}}{s_0^{(m+1)}}\ . \end{aligned}$$

In general, each polynomial $T^{n-1}g(z)$ has lower degree than the polynomial $T^ng(z)$.

From these definitions, it follows that *g*(*z*) and $\tilde{g}(z)$ having a root $z_1=e^{i\theta }$ on the unit circle implies either $Tg(z_1) = \widetilde{Tg}(z_1) = 0$ or $g(0)=0$ (giving undefined *Tg*(*z*)). By iteration, for all *n*, either $T^ng(z_1) = 0$ or $T^{n-1}g(0) = 0$ so that $T^ng(z)$ is undefined.

Firstly, consider the case where $T^ng(z)$ is first undefined for some $n=p+1$, where $0<p \le d-1$.^9^ This imposes that

140 $$\begin{aligned} T^{p}g(0)=s_0^{(d+1-p)}=0\implies \left( s_0^{(d+2-p)}\right) ^2=\left( s_{d+1-p}^{(d+2-p)}\right) ^2\ . \end{aligned}$$

Thus,

141 $$\begin{aligned} |b_{d+1-p}|= \left| \frac{s_{d+1-p}^{(d+2-p)}}{s_0^{(d+2-p)}}\right| = 1\ , \end{aligned}$$

and so any undefined $T^ng(z_1)$ for $0< n\le d$ will give at least one $|b_m|=1$ for $2\le m\le d$.

Now consider the alternate case where $T^ng(z)$ is defined for all $0\le n\le d$. If $z_1 = e^{i\theta }$ is a root of *g*(*z*), then it is a root of all $T^ng(z)$ for all $n\le d$. Thus, the final degree-zero polynomial must satisfy

142 $$\begin{aligned} T^d(z_1) = s_0^{(1)} = 0 \implies \left( s_0^{(2)}\right) ^2=\left( s_{1}^{(2)}\right) ^2\ . \end{aligned}$$

This gives

143 $$\begin{aligned} |b_1| = \left| \frac{s_{1}^{(2)}}{s_0^{(2)}}\right| = 1\ . \end{aligned}$$

Therefore, if *g*(*z*) has a root $z_1 = e^{i\theta }$ on the unit circle, Algorithm 1 must give $|b_m|=1$ for some $1\le m \le d$. As we wish to exclude cases where this occurs, we will only consider polynomials *g*(*z*) without roots on the unit circle. Note, however, that the converse does not hold; having some $|b_k|=1$ does not imply that *g*(*z*) has a root on the unit circle. For example, consider $g(z) = 1-4z+z^2$: this gives $b_2 = 1$, but has roots $z_\pm = 2 \pm \sqrt{3}$ with $|z_\pm | \ne 1$.

## Footnotes

1 Actually, the $N=2$ model we consider differs by $A_k \mapsto (-1)^{k+1} A_k$ compared to Ref. [19] (where *X* and *Z* are also interchanged). The alternating sign can be toggled by the unitary transformation $\prod _j Z_j$ and so we can draw the same conclusion.

2 The state $|\varphi \rangle$ is a series of finite-bond dimension MPOs $\textrm{M}^{(k)}$ operating on an MPS $|\psi _p\rangle$, and so it must itself be an MPS of finite bond dimension. Upper bounds on the bond dimension are straightforward to find, since the $A_m$ have support on $|m|+1$ sites. For example, for $p=0$, an upper bound on the bond dimension $\chi$ is $\log _N\chi =\frac{1}{2}d(d+3)$. Importantly, this does not depend on system size. For the $N=2$ case, optimal bounds on the bond dimensions can be found in certain cases, see discussion in Refs. [19, 20].

3 While this is not the case in the clock models for odd *p*, we actually only require that the ground state eigenspace of $A_p$ has a basis that diagonalises $A_\alpha +G_\alpha$ for all $\alpha$. While guaranteed if the ground state is unique (as for even *p*), a suitable basis can also be constructed for odd *p* in the clock models, as discussed in Appendix D.

4 $\mathcal {W}$ is only defined for $L = 0 \mod N$. However, for any chain length, we can show $(A_1+G_1) |\psi _{0}\rangle = 0$ by explicitly evaluating over the lattice.

5 Note that we evaluate the one-point function for simplicity, ignoring issues with the left boundary. A calculation of the two-point correlation function $\langle \mu _0\mu _k\rangle$ would avoid such issues, and can be evaluated similarly, leading to the square of Eq. (41).

6 Note that a slight modification must be made if $\mathfrak {h}(z)$ has repeated roots. As noted in Ref. [30], it is not necessary to consider this case for the clock models.

7 Generally speaking, computing eigenvalues from functional equations does not give information about their multiplicity, which can be zero [29].

8 For example, for $N=2$, we have $A_l = \pm A_{l+L}$ and $G_l = \pm G_{l+L}$. These terms could cause cancellations which would not occur in models with $N>2$, leading us to conclude the wrong general form of $\{a_\alpha , g_\alpha \}$. We will argue that such cancellations do not occur in our calculations.
